# Alveolin proteins in the *Toxoplasma* inner membrane complex form a highly interconnected structure that maintains parasite shape and replication

**DOI:** 10.1371/journal.pbio.3002809

**Published:** 2024-09-12

**Authors:** Peter S. Back, Vignesh Senthilkumar, Charles P. Choi, Justin J. Quan, Qing Lou, Anne K. Snyder, Andrew M. Ly, Justin G. Lau, Z. Hong Zhou, Gary E. Ward, Peter J. Bradley

**Affiliations:** 1 Molecular Biology Institute, University of California, Los Angeles, Los Angeles, California, United States of America; 2 Department of Microbiology, Immunology, and Molecular Genetics, University of California, Los Angeles, Los Angeles, California, United States of America; 3 Department of Materials Science and Engineering, University of California, Los Angeles, Los Angeles, California, United States of America; 4 Department of Microbiology and Molecular Genetics, Larner College of Medicine, University of Vermont, Burlington, Vermont, United States of America; University of South Florida, UNITED STATES OF AMERICA

## Abstract

Apicomplexan parasites possess several specialized structures to invade their host cells and replicate successfully. One of these is the inner membrane complex (IMC), a peripheral membrane-cytoskeletal system underneath the plasma membrane. It is composed of a series of flattened, membrane-bound vesicles and a cytoskeletal subpellicular network (SPN) comprised of intermediate filament-like proteins called alveolins. While the alveolin proteins are conserved throughout the Apicomplexa and the broader Alveolata, their precise functions and interactions remain poorly understood. Here, we describe the function of one of these alveolin proteins in *Toxoplasma*, IMC6. Disruption of IMC6 resulted in striking morphological defects that led to aberrant invasion and replication but surprisingly minor effects on motility. Deletion analyses revealed that the alveolin domain alone is largely sufficient to restore localization and partially sufficient for function. As this highlights the importance of the IMC6 alveolin domain, we implemented unnatural amino acid photoreactive crosslinking to the alveolin domain and identified multiple binding interfaces between IMC6 and 2 other cytoskeletal IMC proteins—IMC3 and ILP1. This provides direct evidence of protein–protein interactions in the alveolin domain and supports the long-held hypothesis that the alveolin domain is responsible for filament formation. Collectively, our study features the conserved alveolin proteins as critical components that maintain the parasite’s structural integrity and highlights the alveolin domain as a key mediator of SPN architecture.

## Abstract 2

Picomplexan parasites require specialized structures like the inner membrane complex to invade host cells and replicate. This study shows that the alveolin protein IMC6 is crucial for maintaining cellular morphology, invasion and successful replication in Toxoplasma gondii.

## Introduction

The superphylum Alveolata contains a remarkably diverse group of protozoans, including the free-living ciliates and dinoflagellates as well as the parasitic apicomplexans. Despite these lifestyle differences, the alveolates are unified by a peripheral system of membranes underlying the plasma membrane called the alveoli and a conserved group of proteins called the alveolins [1]. These 2 features define the superphylum and highlight the alveoli as critical cellular structures for the survival of these organisms. Of the alveolates, the parasitic apicomplexans have garnered the most attention due to their severe public health and economic burdens [2]. Notable human pathogens include *Toxoplasma gondii* (toxoplasmosis), *Plasmodium* spp. (malaria), and *Cryptosporidium* spp. (cryptosporidiosis), which together cause an enormous disease burden globally that results in a tremendous number of fatalities [3–5]. Veterinary pathogens include *Neospora* spp. and *Eimeria* spp., which cause large numbers of disease in livestock and subsequent economic losses [6,7].

In the Apicomplexa, the alveoli are called the inner membrane complex (IMC) and are situated underneath the plasma membrane as in other alveolates. The IMC has been studied most extensively in *T*. *gondii* and *Plasmodium* spp. and contains 3 main functions. It first serves as a platform for the glideosome, an actin-myosin motor that powers gliding motility and host cell invasion [8]. Second, it provides a scaffold for daughter cell assembly throughout the replication process [9–11]. Finally, the apical cap region of the IMC houses the regulatory center for cytoskeletal disassembly during the final stages of replication [12–14]. While these functions are generally conserved in other apicomplexans, it is unlikely that they are conserved in other alveolates. Due to their nonparasitic lifestyle, ciliates and dinoflagellates have likely co-opted their respective alveoli to meet the demands of a free-living environment. Thus, determining the precise roles of the alveolin proteins promises to provide insights into these differences.

In apicomplexans, the alveolins are believed to be the major constituents of the subpellicular network (SPN) [12,13,15–21]. The SPN is a highly interwoven mesh of filaments that underlies the membrane vesicles of the IMC and provides a cytoskeletal foundation for the parasite [9,18]. The alveolins are categorized by the presence of a conserved alveolin domain, a region of the protein containing proline and valine-rich repeats [1]. It is speculated that the alveolin domain mediates the formation of filaments via protein–protein interactions that ultimately establish the SPN. However, direct experimental evidence is lacking, largely due to the detergent insoluble nature of these proteins that limit the use of standard interaction methods such as co-immunoprecipitation (co-IP). To overcome this barrier, we recently adapted unnatural amino acid (UAA) photocrosslinking to *T*. *gondii*, which identifies interacting partners within the native environment of the parasite [22]. This approach uses the zero-length crosslinker *p*-azidophenylalanine (Azi), enabling us to map specific binding interfaces that provide structural information regarding the interaction [23]. We previously used this technique to determine that TgILP1 binds 2 alveolin proteins, IMC3 and IMC6. However, these interactions were mapped to their variable N- and C-terminal regions rather than the core alveolin domains. Thus, the alveolin domains remain unexplored in this family of proteins. Functionally, only some of the *T*. *gondii* alveolin proteins have been studied so far. Of these, many were shown to play a role in replication or in providing tensile strength, but none were shown to be important or essential for parasite fitness [24,25]. In contrast, the alveolins that have eluded characterization are typically those with low genome-wide CRISPR screen (GWCS) phenotype scores, suggesting essentiality [26].

In this study, we report the successful knockout of the *T*. *gondii* alveolin (TgIMC6). We determine its critical role in maintaining parasite shape and describe the downstream consequences on parasite motility and host cell invasion. We also characterize the severe replication errors exhibited by Δ*imc6* parasites. To determine the significance of the alveolin domain, we use deletion analyses to demonstrate that the alveolin domain is largely sufficient to restore IMC6 localization and function. We then implement the recently developed photoreactive crosslinking approach to pinpoint direct interactions and map the binding interfaces between the IMC6 alveolin domain and other cytoskeletal proteins of the SPN. This firmly establishes the alveolin domain as a major region of protein–protein contact.

## Results

### Disrupting IMC6 causes severe defects in vitro and in vivo

**Fig 1:**
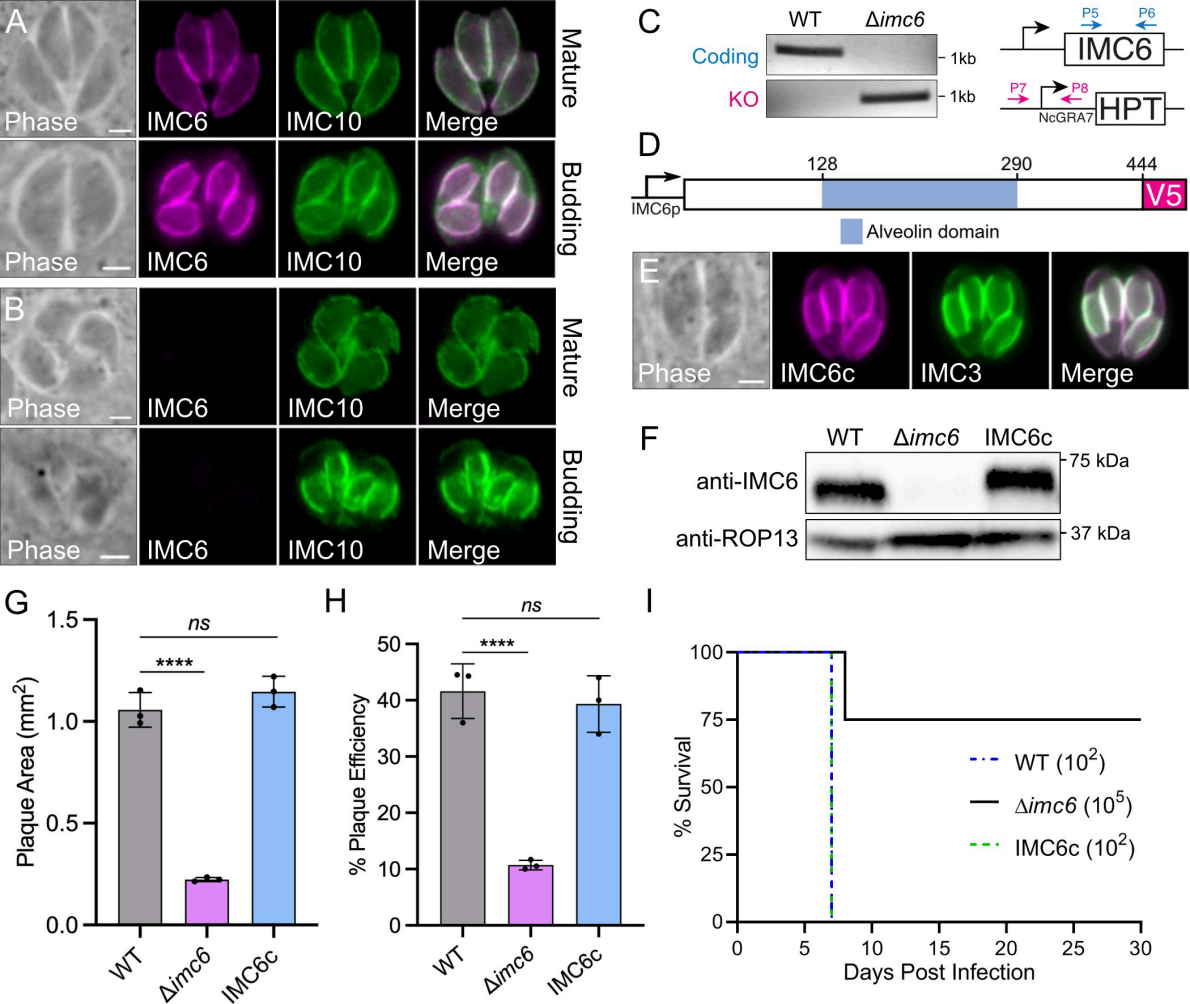
Disrupting IMC6 causes severe fitness and virulence defects. (**A**) IFA of WT parasites showing proper IMC6 localization in mature and budding parasites. (**B**) IFA of Δ*imc6* parasites showing absence of IMC6. (**C**) PCR verification of the genomic loci from WT (RHΔ*hxgprt*) and Δ*imc6* parasites. Diagram illustrates primers used to amplify the IMC6 coding sequence (blue arrows) and the site of recombination for the knockout locus (magenta arrows). (**D**) Diagram of the full-length complementation construct, which includes the endogenous promoter and a V5 epitope tag. Full-length protein is 444 amino acids, with aa128-290 encompassing the alveolin domain. (**E**) IFA of IMC6c parasites showing restored localization of IMC6 (anti-V5). (**F**) Western blot of whole cell lysates depicting the absence (Δ*imc6*) and rescue (IMC6c) of IMC6 expression. The difference in migration between WT and IMC6c is due to the V5 epitope tag in the complemented version. ROP13 was used as a loading control. (**G**) Quantification of plaque areas, where each point represents a biological replicate with 30–40 plaques measured per replicate. Data are plotted as the mean ± SD, and significance was determined using two-way ANOVA. ****: *p* < 0.0001. (**H**) Quantification of plaque efficiency, measured by counting the number of plaques relative to the total number of parasites added. Data are plotted as the mean ± SD, and significance was determined using multiple two-tailed *t* tests. ****: *p* < 0.0001. (**I**) Survival curve of mice (*n* = 4) injected with the indicated parasite strain. All scale bars are 2 μm. The raw data underlying this figure can be found in S1 Data. IFA, immunofluorescence assay; KO, knockout; WT, wild-type.

Of the alveolin proteins with low phenotype scores, IMC6 was assigned the highest score of −3.19 [26,27]. Recent work from our lab demonstrated that genes with similar phenotype scores (ISAP1: −3.49 and IMC29: −3.95) could be disrupted [28,29]. Thus, we wanted to determine if IMC6 could also be knocked out. As previously reported, IMC6 localizes to the IMC of both maternal and daughter parasites with enrichment in the daughter buds (Fig 1A) [18]. We were indeed successful in generating a knockout strain (Δ*imc6*), verified by the absence of protein expression in immunofluorescence assays (IFA) and by recombination at the genomic locus using PCR (Fig 1B and 1C). We then generated a complementation construct with the full-length IMC6 cDNA driven by its endogenous promoter with a C-terminal 1xV5 epitope tag (Fig 1D). Expressing this construct in Δ*imc6* parasites restored protein localization and expression similar to wild-type levels as determined by IFA and western blot (complemented strain: IMC6c; Fig 1E and 1F).

To assess the overall fitness cost of disrupting *IMC6*, we first performed plaque assays. Measuring both plaque area and plaque-forming efficiency indicated a severe 78.8% reduction in plaque size and a 71.7% reduction in plaque efficiency (Fig 1G and 1H). These defects were fully rescued in the IMC6c parasites. To determine if virulence is also affected, we infected mice with 10^2^ wild-type, 10^5^ Δ*imc6*, or 10^2^ IMC6c parasites (Fig 1I). As expected, the mice injected with wild-type or IMC6c parasites succumbed to the infection after 7 days. In contrast, of the 4 mice injected with a 1,000-fold greater number of knockout parasites, 3 survived the infection. This demonstrates a dramatic decrease in virulence that corroborates the in vitro growth defects and highlights the importance of IMC6 for parasite fitness.

### Disruption of IMC6 causes extreme shape defects

**Fig 2:**
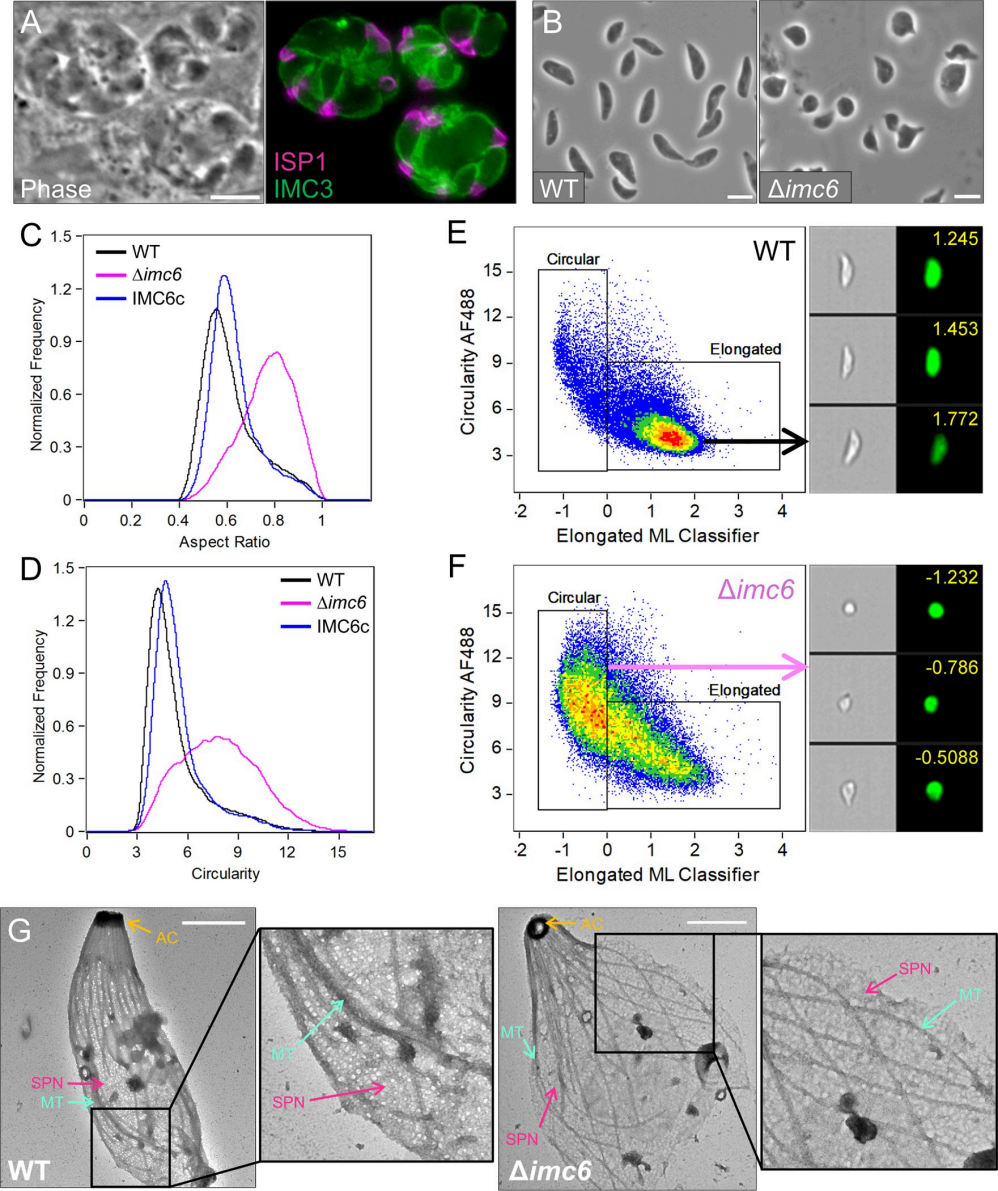
Δ*imc6* parasites are extremely misshapen. (**A**) Phase contrast and IFA of a representative field of Δ*imc6* vacuoles. ISP1 stains the apical end, while IMC3 stains the body portion of the IMC. Scale bar is 5 μm. (**B**) Phase contrast field of WT and Δ*imc6* extracellular parasites. Scale bar is 5 μm. (**C**) ImageStream analysis of the aspect ratios for each population. Greater aspect ratios indicate rounder objects. Median aspect ratio for WT is 0.59, Δ*imc6* is 0.78, and IMC6c is 0.61. (**D**) ImageStream analysis using the circularity parameter, where greater values indicate rounder objects. Median circularity value for WT is 4.72, Δ*imc6* is 7.78, and IMC6c is 5.05. The raw data underlying these medians can be found in 10.5281/zenodo.13333981. (**E**, **F**) Bivariate plots of the Circularity feature vs. the Elongated ML classifier on the WT (**E**) and Δ*imc6* (**F**) samples. The WT population contains 85.3% elongated and 13.3% circular objects. The Δ*imc6* population contains 46.6% elongated and 46.5% circular objects. This population shift is highlighted by the density plots. The assigned values indicate confidence given by the Elongated ML classifier, where higher values indicate greater confidence of elongatedness. (**G**) TEM of detergent-extracted and negatively stained parasites. Insets are zoomed in 3-fold. Scale bar is 1 μm. AC, apical complex; IFA, immunofluorescence assay; IMC, inner membrane complex; ISP1, IMC sub-compartment protein 1; ML, Machine Learning; MT, microtubules; SPN, subpellicular network; TEM, transmission electron microscopy; WT, wild-type.

Upon disrupting *IMC6*, one of the most striking changes was parasite morphology. Δ*imc6* parasites appeared grossly misshapen in intracellular vacuoles, stained with the IMC markers IMC sub-compartment protein 1 (ISP1), which labels the apical end, and IMC3, which labels the body (Fig 2A). Extracellular Δ*imc6* parasites were similarly misshapen, as assessed by phase contrast microscopy (Fig 2B). To quantify the shape defects in a nonbiased manner, we used ImageStream imaging flow cytometry [21]. We measured >20,000 individual extracellular parasites for each strain and evaluated parasite morphology under 2 main categories—aspect ratio and circularity (Fig 2C and 2D). As expected, the aspect ratios of wild-type parasites indicated an elongated morphology with a median of 0.59. In contrast, the aspect ratios of knockout parasites were higher with a median of 0.78, indicating a substantially rounder shape on average. This shape defect was fully restored in the complemented parasites. Similarly for circularity, the wild-type and IMC6c populations contained, on average, less circular cells with medians of 4.7 and 5.1, respectively. In contrast, the circularity values for Δ*imc6* parasites indicated substantially rounder cells with a median of 7.8, again highlighting the grossly misshapen morphology of the knockout parasites.

We extended this quantification by supplementing the circularity measurement with the Elongated Machine Learning (EloML) classifier. The EloML classifier considers a set of 10 features and assigns a binary classification for each object as either elongated or circular (S1 Fig). Taught on 200 wild-type and 200 knockout parasites, the EloML classifier was designed to create a threshold for elongated/circular objects and to assign a confidence value for each one. Upon analyzing our parasites with this approach, the wild-type strain contained 85.3% elongated and 13.3% circular populations (Fig 2E). In contrast, the Δ*imc6* strain contained 46.6% elongated and 46.5% circular populations (Fig 2F). This dramatic shift in the population is illustrated by the density scatter plots. Taken together, imaging flow cytometry demonstrates that IMC6 is critical to maintain the parasite’s shape.

To determine if the shape defects are caused by structural damage to the SPN filaments, we performed transmission electron microscopy (TEM) on detergent-extracted parasites. We found that the parasite ghosts generally resemble the shapes of whole extracellular parasites—elongated for wild-type and swollen for knockout (Fig 2G, additional images in S2 Fig). Importantly, we found that the SPN and microtubule array are clearly present and generally intact in the knockout parasites, indicating that they are not overly sensitive to detergent extraction. Thus, while subtle differences may exist, the cytoskeleton of Δ*imc6* parasites appears to remain largely intact despite the severe shape defects.

### Δ*imc6* parasites exhibit altered motility and less efficient invasion

**Fig 3:**
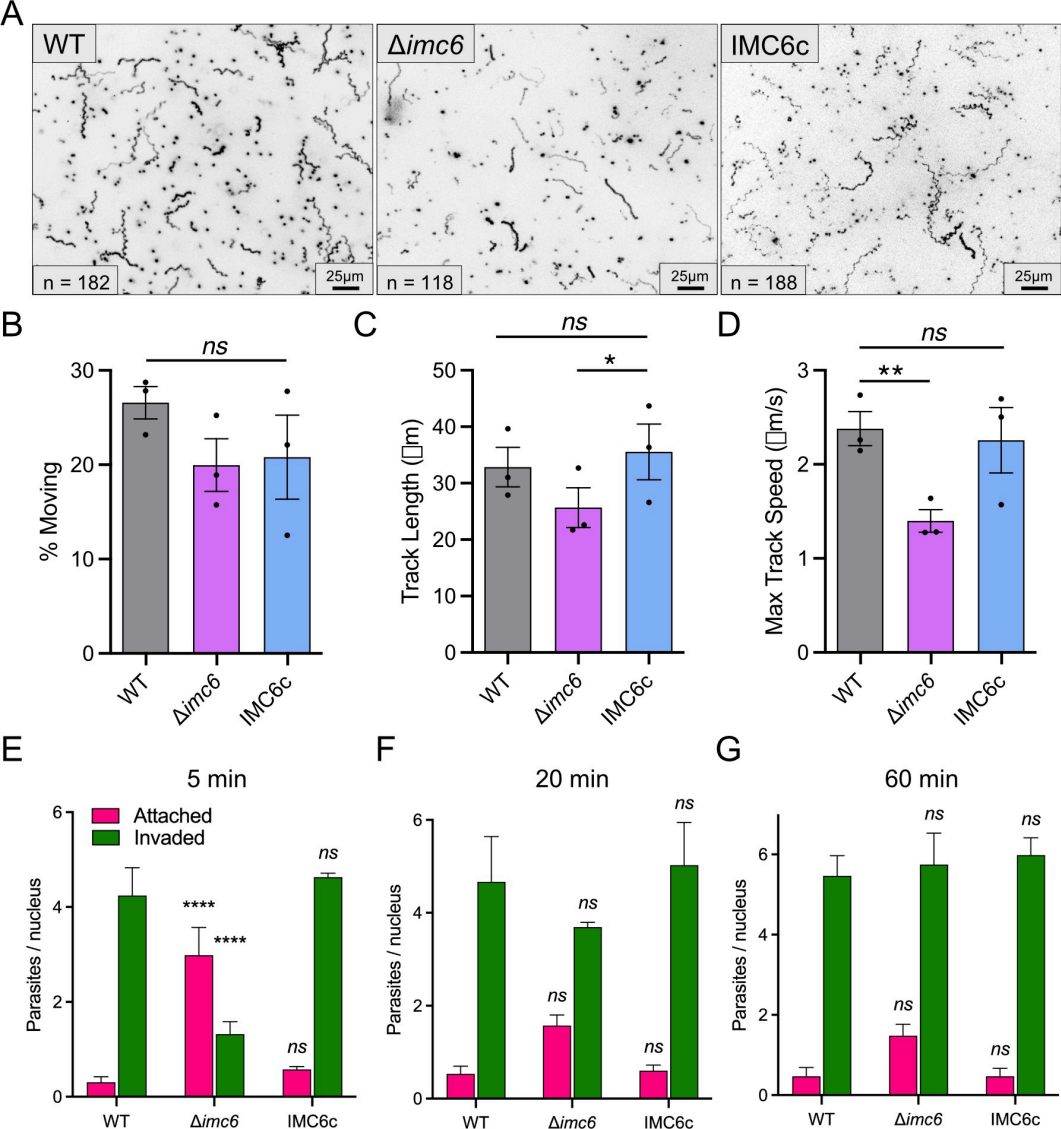
Δ*imc6* parasites are motile but invade less efficiently. (**A**) Representative maximum intensity projections showing trajectories of parasites in Matrigel, imaged over 80 seconds. Greyscale images were inverted to better visualize trajectories. n indicates the number of tracks quantified for the technical replicates shown. Scale bars are 25 μm. (**B**-**D**) Quantifications of percent moving, track length, and maximum track speed. All trajectory data were acquired from 3 biological replicates, each consisting of 3 technical replicates. Data are plotted as the mean ± SEM, and significance was determined using two-way ANOVA. *: *p* = 0.0479. **: *p* = 0.0340. (**E**-**G**) Red-green invasion assays with 5-minute (**E**), 20-minute (**F**), and 60-minute (**G**) invasion permissive conditions. Magenta represents extracellular/attached parasites, and green represents invaded parasites. Triplicate experiments were performed by counting the number of parasites per host nucleus. Significance was calculated using two-way ANOVA. ****: *p* < 0.0001. The raw data underlying this figure can be found in S1 Data.

As parasite morphology has been shown to affect gliding motility, we evaluated Δ*imc6* parasites using 3D motility assays [30]. Fig 3A shows representative maximum intensity projections (MIPs) of parasite motility in the Matrigel-based environment, illustrating the proportion of motile parasites, track length, and track shape. To quantify these parameters, motility images were captured over an 80-second period. Using a 2.8-μm minimum displacement threshold (see Materials and methods), we observed no significant difference in the proportion of parasites moving or track length between wild-type and knockout parasites (Fig 3B and 3C). However, the Δ*imc6* parasites exhibited a modest but significant defect in maximum speed achieved along their trajectories compared to wild-type parasites (Fig 3D). We also noted differences in trajectory shape. For wild-type parasites, the representative MIPs and videos depict the characteristic asymmetrical helical shape of the trajectory (Fig 3A and S1 and S2 Videos) [30]. Many of the Δ*imc6* trajectories, however, show reduced helicity, ranging from no apparent helicity to a helix with a much longer period (i.e., a “flatter” corkscrew shape; Fig 3A and S3–S6 Videos). We were unable to quantify these differences in shape due to the inconsistencies in the Δ*imc6* tracks. Nevertheless, both the decrease in maximum trajectory speed and differences in trajectory shape were reversed in the complemented strain, as was a small but nonsignificant difference in track length (Fig 3A–3D and S7 and S8 Videos). Together, these assays indicate that motility is altered in the Δ*imc6* parasites. To assess whether these motility differences are due to problems in microneme secretion, we performed a microneme secretion assay and found that exocytosis remains unperturbed in the Δ*imc6* parasites (S3A Fig). This suggests that the differences in motility are primarily caused by the shape differences.

Since motility is closely linked to invasion and egress, we wanted to determine if the altered motility is associated with a change in invasion and egress efficiency. We first performed a standard invasion assay that allows parasites to invade for 5 minutes. With this, the number of Δ*imc6* parasites invaded (1.3 ± 0.3 parasites/host cell) was significantly fewer compared to that of wild-type parasites (4.2 ± 0.6 parasites/host cell), while the total number of parasites attached and invaded remained comparable (Fig 3E). Thus, the ability to attach to a host cell appears unaffected, but the efficiency of invasion is severely hampered. We hypothesized that the slower speeds observed in the 3D motility assay may be compensated given more time. To test this, we increased the time spent in invasion-permissive conditions to 20 and 60 minutes (Fig 3F and 3G). Consistent with our hypothesis, the number of invaded Δ*imc6* parasites increased significantly at the 20-minute and even more at the 60-minute time points (3.7 ± 0.1 and 5.8 ± 0.8 parasites/host cell, respectively). This demonstrates that the knockout parasites can largely catch up to the invasion efficiency of wild-type parasites given enough time. To determine if egress is also affected, we performed an induced egress assay and found no difference between wild-type and knockout parasites (S3B Fig). Thus, Δ*imc6* parasites exhibit modest defects in gliding motility and significantly slower invasion but no impairment in egress.

### IMC6 is important for proper parasite replication

**Fig 4:**
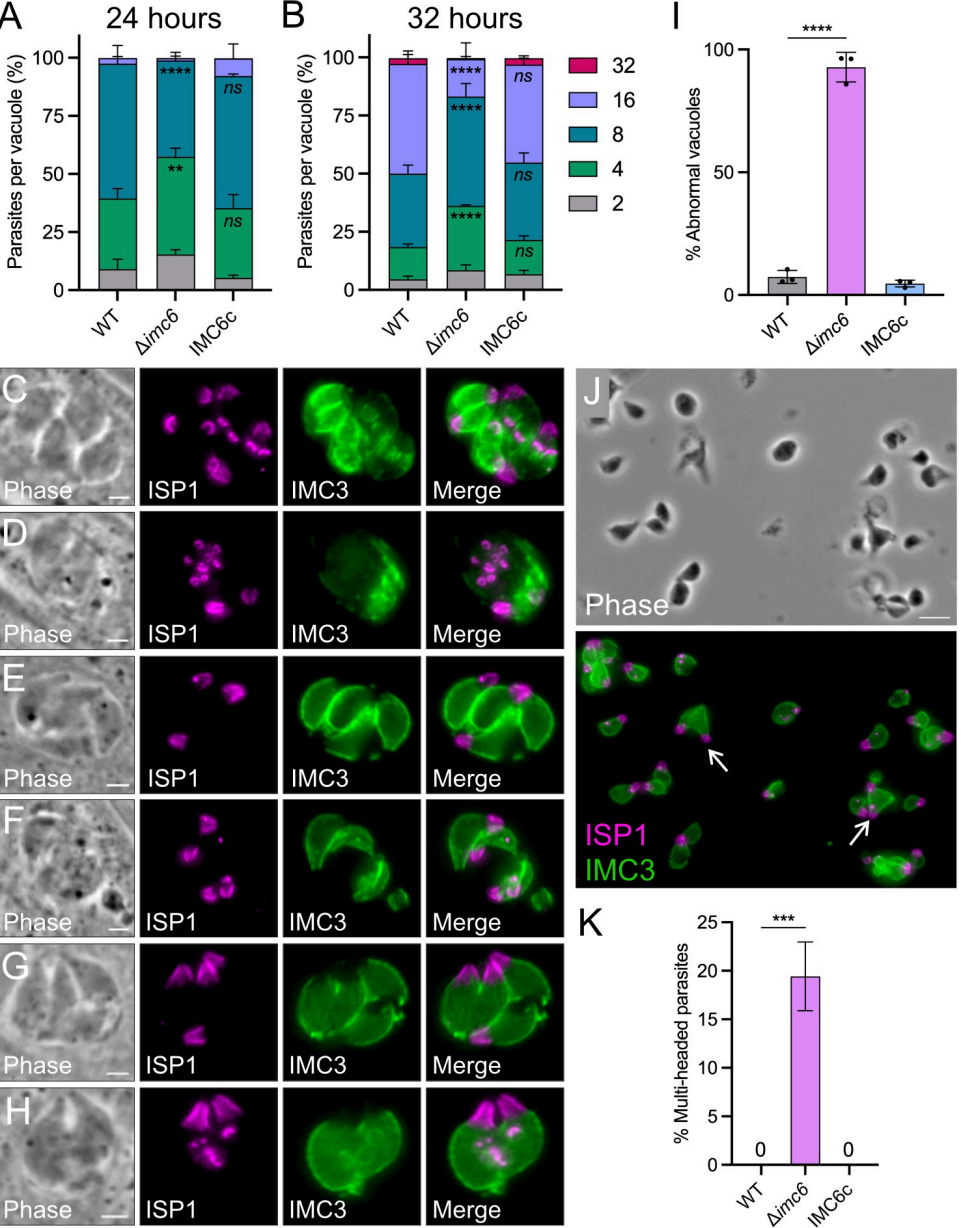
IMC6 is important for proper endodyogeny. (**A**, **B**) Replication assays conducted at 24 (**A**) and 32 (**B**) hpi. Each category represents the number of parasites per vacuole. Triplicate experiments were performed by quantifying >300 total vacuoles across at least 15 different fields per replicate. Data are plotted as the mean ± SD, and significance was calculated using two-way ANOVA. (**A**) shows significance for 4 and 8 parasites per vacuole. (**B**) shows significance for 4, 8, and 16 parasites per vacuole. **: *p* = 0.0018. ****: *p* < 0.0001. (**C**-**H**) Representative IFAs depicting various replication defects, including asynchrony (**C**), multi-daughters (**D**), disorganization (**E**), breaks in IMC structure (**F**), and incomplete separation in mature (**G**) and budding (**H**) parasites. All IFAs were conducted about 30 hpi. All scale bars are 2 μm. (**I**) Quantification of abnormal vacuoles as defined by the presence of any one of the replication defects. Triplicate experiments were performed with >200 total vacuoles counted across at least 15 different fields per replicate. Data are plotted as the mean ± SD, and significance was calculated using two-tailed *t* tests. ****: *p* < 0.0001. (**J**) Phase contrast and IFA of a field of extracellular Δ*imc6* parasites. White arrows highlight incompletely separated parasites. Scale bar is 5 μm. (**K**) Quantification of the incomplete separation phenotype in extracellular parasites. Triplicates performed by counting >300 individual parasites across at least 15 different fields per replicate. Data are plotted as the mean ± SD, and significance was calculated using two-tailed *t* tests. ****: *p* < 0.0001. The raw data underlying this figure can be found in S1 Data. hpi, hours post infection; IFA, immunofluorescence assay; IMC, inner membrane complex.

As the subtle defects in motility and invasion are likely insufficient to produce the severe plaque defects of Δ*imc6* parasites, we evaluated whether replication is affected since the localization of IMC6 is enriched in daughter buds. We first assessed overall replication by quantifying the number of parasites per vacuole at 24 and 32 hours postinfection (hpi) (Fig 4A and 4B). At 24 hpi, the majority of wild-type vacuoles contained 8 parasites. In contrast, Δ*imc6* vacuoles were more evenly split between 4 and 8 parasites per vacuole, indicating they are progressing significantly slower compared to wild-type parasites. This defect became more pronounced at 32 hpi, where the majority of wild-type vacuoles contained 16 parasites, while a significantly fewer number of Δ*imc6* vacuoles contained 16 (Fig 4B). Thus, Δ*imc6* parasites can progress through endodyogeny, albeit at a considerably slower rate than wild-type parasites.

To pinpoint specific replication defects, we first stained Δ*imc6* parasites with a series of markers to evaluate various parasite organelles. These IFAs indicated that the apicoplast, mitochondria, plant-like vacuole (PLV/VAC), micronemes, and rhoptries localize properly and appear unaffected in the absence of IMC6 (S4 Fig). We then stained parasites with the IMC markers ISP1 and IMC3 to assess the fidelity of endodyogeny. We noticed several errors including asynchronous division, >2 daughter buds per maternal parasite, grossly unorganized vacuoles, large breaks in the IMC structure, and incomplete daughter separation (Fig 4C–4H). Many of the Δ*imc6* vacuoles exhibited multiple replication defects simultaneously. Thus, rather than quantifying each defect individually, we categorized each vacuole as either standard or abnormal depending on the presence of any one of these replication defects. This revealed an astonishing 92.8 ± 6.1% of abnormal Δ*imc6* vacuoles compared to 7.4 ± 2.7% of abnormal wild-type vacuoles and 4.7 ± 1.4% of abnormal IMC6c vacuoles (Fig 4I).

We were particularly intrigued by the incompletely separated parasites. During the final stages of endodyogeny, the parasites seem to stall very early during cytokinesis, resulting in conjoined bodies (Fig 4G). We even observed conjoined parasites that have begun the next round of division, suggesting that cytokinesis is decoupled from the cell cycle in these parasites (Fig 4H). This defect was also seen in naturally egressed extracellular parasites, with 19.4 ± 3.5% of Δ*imc6* parasites exhibiting this phenotype (Fig 4J and 4K). To determine if this is linked to basal complex formation, we endogenously tagged BCC1 in both wild-type and knockout parasites [31,32]. First, we assessed properly separated parasites and found that BCC1 localizes to the basal ends of each mature and daughter parasite (S5A and S5B Fig). Moreover, the maternal basal complexes appear to be fully contracted, indicating that both the formation and contraction of the basal complex are unaffected in knockout parasites. We then evaluated the basal complex in incompletely separated parasites, which again showed proper BCC1 localization in mature and daughter cells (S5C Fig). This indicates that the cytokinesis phenotype is not due to basal complex defects. Taken together, the severe defects in replication combined with slower invasion likely explain the drastic growth defects in vitro and virulence defects in vivo.

### The alveolin domain is largely sufficient for IMC6 localization and partially sufficient for its function

**Fig 5:**
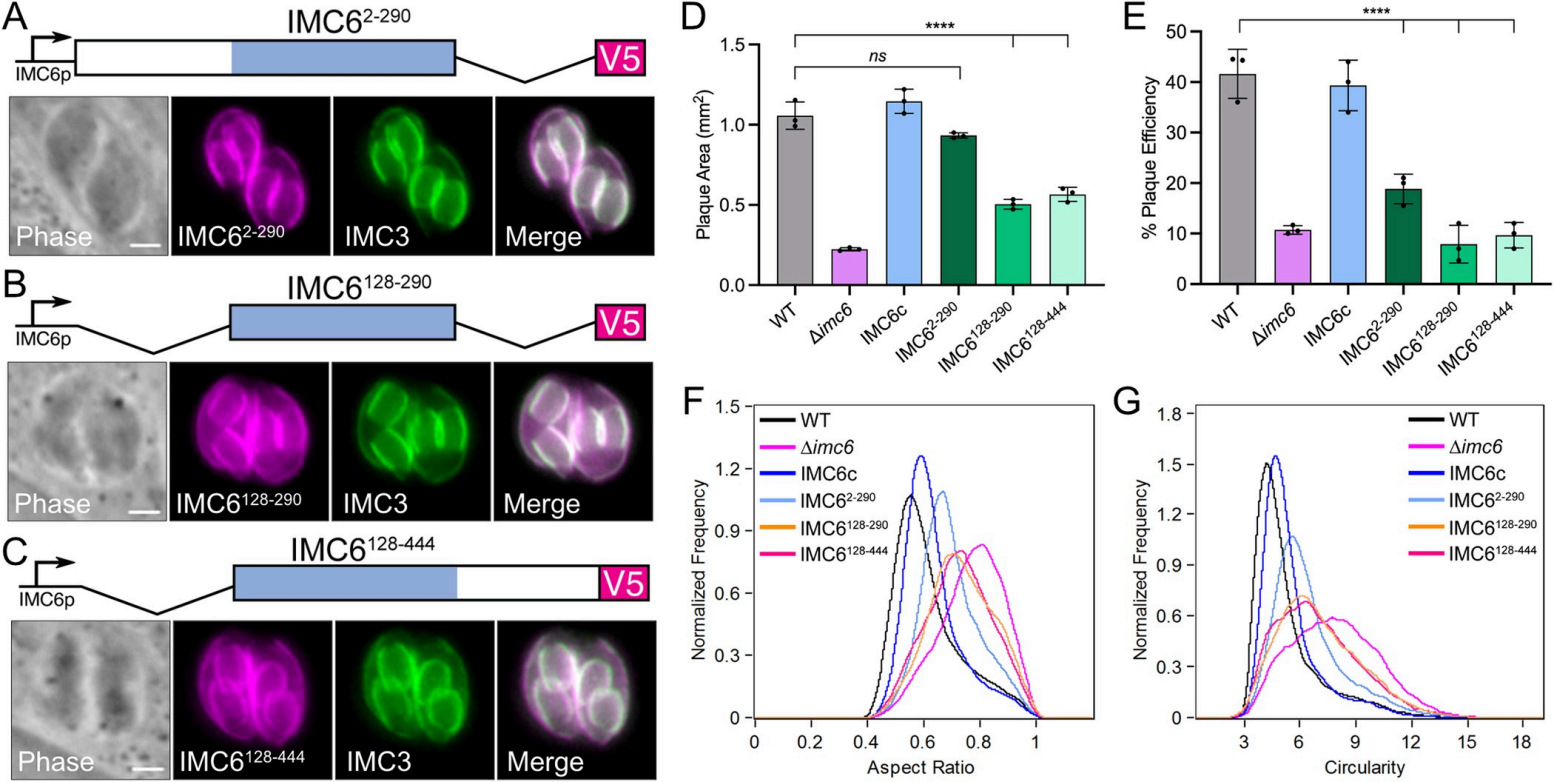
Domain analysis of IMC6 correlates parasite form and function. (**A**) Diagram and IFA of IMC6^2-290^, showing proper localization. (**B**) Diagram and IFA of IMC6^128-290^, showing partial IMC localization with additional cytoplasmic staining. (**C**) Diagram and IFA of IMC6^128-444^, showing partial IMC localization with additional cytoplasmic staining. (**D**) Quantification of plaque areas for each deletion construct. The data for WT, Δ*imc6*, and IMC6c were collected in the same experiment as those from Fig 1G and are shown here again to facilitate a direct comparison. Triplicates were performed by measuring 30–40 plaques per replicate, data are plotted as the mean ± SD, and significance was calculated using two-way ANOVA. ****: *p* < 0.0001. (**E**) Quantification of plaque efficiency. The data for WT, Δ*imc6*, and IMC6c were collected in the same experiment as those from Fig 1H and are shown here again to facilitate a direct comparison. Data are plotted as the mean ± SD, and significance was calculated using two-tailed *t* tests. ****: *p* < 0.0001. (**F**, **G**) ImageStream analysis of each population, showing aspect ratio (**F**) and circularity (**G**). The data for WT, Δ*imc6*, and IMC6c were collected in the same experiment as those from Fig 2C and 2D and are shown here again for ease of comparison. Median aspect ratio for IMC6^2-290^ is 0.68, IMC6^128-290^ is 0.73, and IMC6^128-444^ is 0.73. Median circularity value for IMC6^2-290^ is 6.04, IMC6^128-290^ is 6.63, and IMC6^128-444^ is 6.71. All scale bars are 2 μm. The raw data underlying these medians can be found in 10.5281/zenodo.13333981, and the raw data underlying the graphs can be found in S1 Data. IFA, immunofluorescence assay; IMC, inner membrane complex; WT, wild-type.

We and others have previously reported that the alveolin domain is largely sufficient for the proper localization of IMC3, IMC6, and IMC8 [18,22]. However, these studies were done in wild-type parasites, so the functional significance of the alveolin domain has not been tested. Thus, we generated IMC6 deletion constructs that truncate the N-terminal and/or C-terminal regions flanking the alveolin domain and expressed them in Δ*imc6* parasites. We first assessed the localization of each truncated protein and found results consistent with our previous study [22]. The localization of IMC6^2-290^ closely resembles that of the full-length IMC6, while both IMC6^128-290^ and IMC6^128-444^ exhibit slightly more cytoplasmic staining (Fig 5A–5C). This demonstrates that the alveolin domain alone is sufficient to restore much of the protein’s localization, although the N-terminal portion of the protein is also important.

To dissect the function of each domain, we performed plaque assays. Measuring both plaque area and plaque efficiency revealed a pattern that mimics the localization data. IMC6^2-290^ fully rescues the plaque size and partially rescues plaque efficiency. On the other hand, IMC6^128-290^ and IMC6^128-444^ partially rescue plaque size and does not rescue plaque efficiency at all (Fig 5D and 5E). We then used ImageStream to determine if growth defects are linked to parasite shape. Consistent with the plaque assays, the quantifications for aspect ratio and circularity followed a similar pattern (Fig 5F and 5G). Parasites expressing IMC6^2-290^ most resembled the elongated shape of wild-type parasites, though not fully. In contrast, parasites expressing IMC6^128-290^ or IMC6^128-444^ moderately rounded, indicating a partial rescue of shape. Phase contrast images of each complemented strain further support the partial rescue of shape (S6 Fig). Taken together, this domain analysis demonstrates that the alveolin domain alone is largely sufficient for localization and partially sufficient for function, with the N-terminal third of the protein also contributing an important role. We additionally uncover a strong correlation between parasite shape and parasite fitness, with more rounded parasites exhibiting decreased fitness. Thus, IMC6 provides a critical structural role that defines parasite shape, which ultimately impacts parasite motility, invasion, and replication.

### Photoreactive crosslinking reveals numerous contact points in the IMC6 alveolin domain

Along with other alveolin proteins and associated cytoskeletal proteins, IMC6 is believed to provide structural integrity for the parasite by forming a robust network of filaments just underneath the IMC membrane sacs. The prevailing theory is that this SPN is created primarily by protein–protein interactions via the conserved alveolin domains [1,18]. However, direct experimental evidence for this has been lacking, mainly due to the technical difficulty of identifying interactions between detergent-resistant cytoskeletal proteins. To overcome this challenge, we applied UAA photocrosslinking to the IMC6 alveolin domain to directly test whether this domain is involved in mediating the protein–protein interactions.

**Fig 6:**
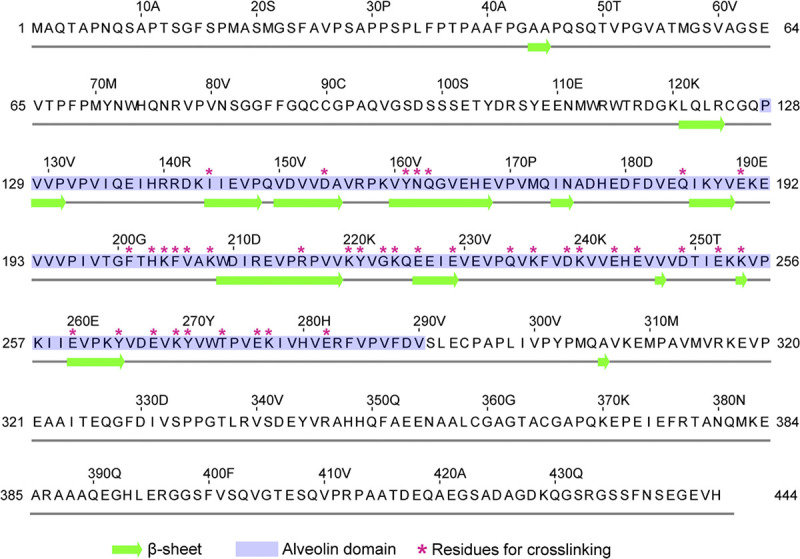
Diagram of the IMC6 sequence and predicted secondary structure. β-sheets are shown in green, the alveolin domain is highlighted in blue, and magenta stars represent residues chosen for crosslinking. Short β-sheets of 1–2 amino acids are unlikely to be real but remain included in the diagram as predicted by JPred4.

**Fig 7:**
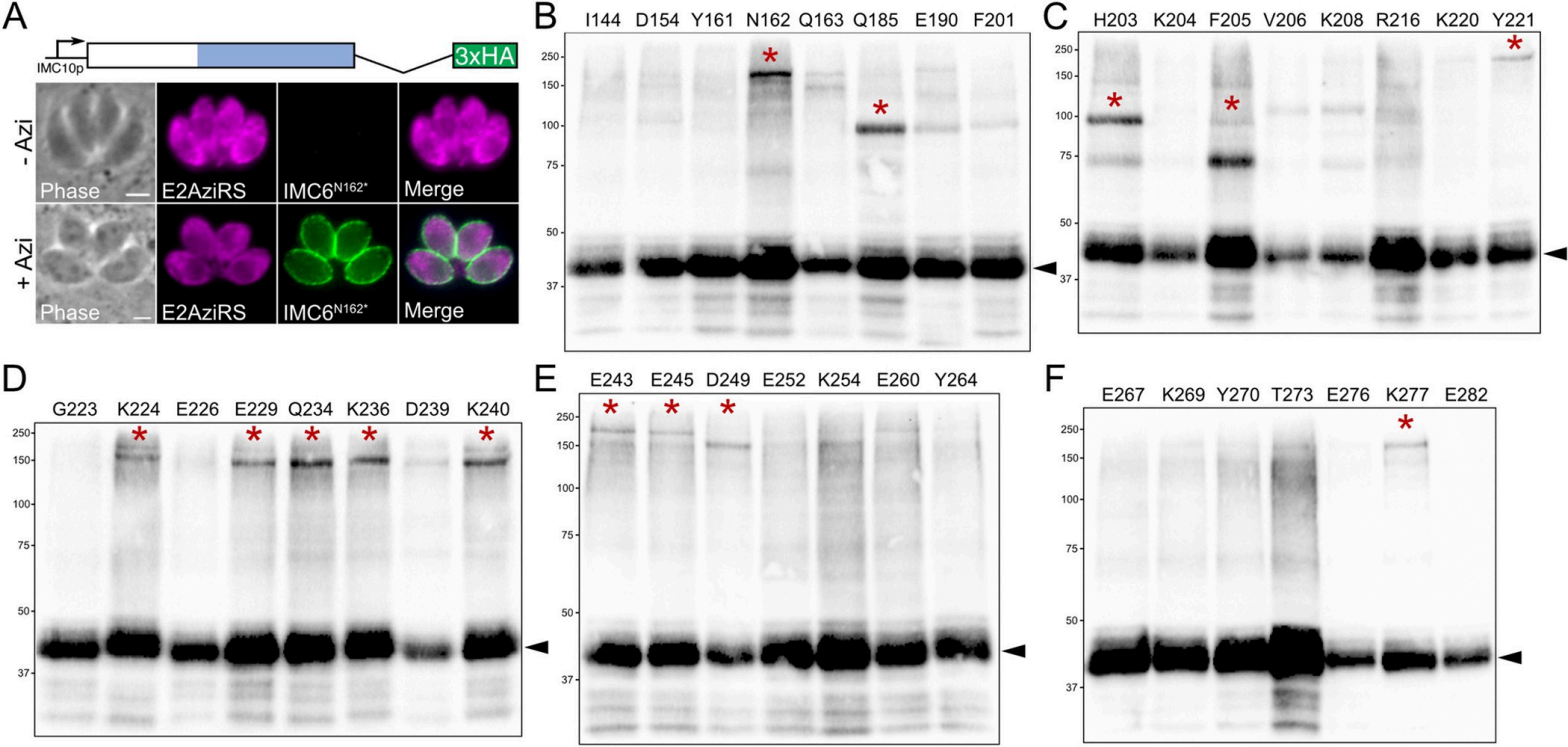
The IMC6 alveolin domain is a major region of protein–protein interactions. (**A**) The complementation construct used for photoreactive crosslinking consists of the IMC6^2-290^ sequence, driven by the IMC10 promoter with a 3xHA epitope tag. IFA of parasites expressing the E2AziRS^3xTy^ (tRNA synthetase), the Azi-specific tandem tRNA cassettes, and the IMC6^2-290^-3xHA construct with N162 mutated to an amber stop codon. Without Azi added to the system, the N162* mutation results in premature stop in translation and no protein expression. Upon Azi addition, Azi is incorporated into the amber stop codon and results in proper expression. Scale bar is 2 μm. (**B**-**F**) Western blot of whole cell lysates following Azi incorporation and UV treatment. Black arrowheads represent uncrosslinked IMC6^2-290^-3xHA that migrates at approximately 45 kDa. Red stars represent crosslinked upshifts that were chosen for further investigation (see S1 Table). All blots were detected with anti-HA. Azi, *p*-azidophenylalanine; HA, hemagglutinin; IFA, immunofluorescence assay.

To determine which residues to mutate into amber stop codons, we prioritized charged residues and those that were bioinformatically determined to be favorable for protein–protein interactions on beta-sheets (see Materials and methods) [33,34]. Using these criteria, we chose 38 residues spanning the alveolin domain (Fig 6). Each amber mutant was constructed with the IMC6^2-290^ sequence as this truncated protein was fully sufficient for localization and its smaller size facilitates the visualization of upshifted crosslinked products by western blot (Fig 5A). The constructs were generated in a complementation vector with a C-terminal 3xHA epitope tag and randomly integrated into parasites equipped with the aminoacyl-tRNA synthetase/tRNA cassettes (Fig 7A) [22]. For each strain expressing an amber mutant, we assessed the incorporation of the UAA (+Azi) and the resulting expression of the mutant protein. We found that none of the amber mutants appeared to affect the localization of the protein; the N162* mutant is shown as a representative example (Fig 7A).

We divided the amber mutants into 5 sets of 7 or 8 residues and subjected them to Azi incorporation and photoreactive crosslinking. Upon analyzing each residue by western blot, we observed a myriad of upshifted products throughout the alveolin domain, each representing a crosslink between IMC6 and an unknown binding partner (Fig 7B–7F). To discern which upshifts to pursue further, we measured the abundance of crosslinked to uncrosslinked product for every residue and only considered residues with >10% ratio (S1 Table). We also excluded residues containing excessive background. Filtering with these criteria resulted in a total of 14 residues. These could be divided into 4 distinct molecular weights, with each group likely representing a different binding partner. Residues Q185, H203, and F205 were crosslinked at both about 75 and 95 kDa, intriguingly with contrasting intensities. Residues N162, Y221, K224, E243, E245, and K277 were crosslinked prominently at about 175 kDa, and residues E229, Q234, K236, K240, and D249 were crosslinked at about 140 kDa. Some of these showed upshifts at both 140 and 175 kDa (K224, E229, Q234, K240, E243, and E245), though with differing intensities. Overall, these crosslinking data demonstrate that the IMC6 alveolin domain contains an abundance of protein–protein interactions with each crosslinked product representing a binding interface.

### IMC3 binds IMC6 at multiple residues across the alveolin domain

**Fig 8:**
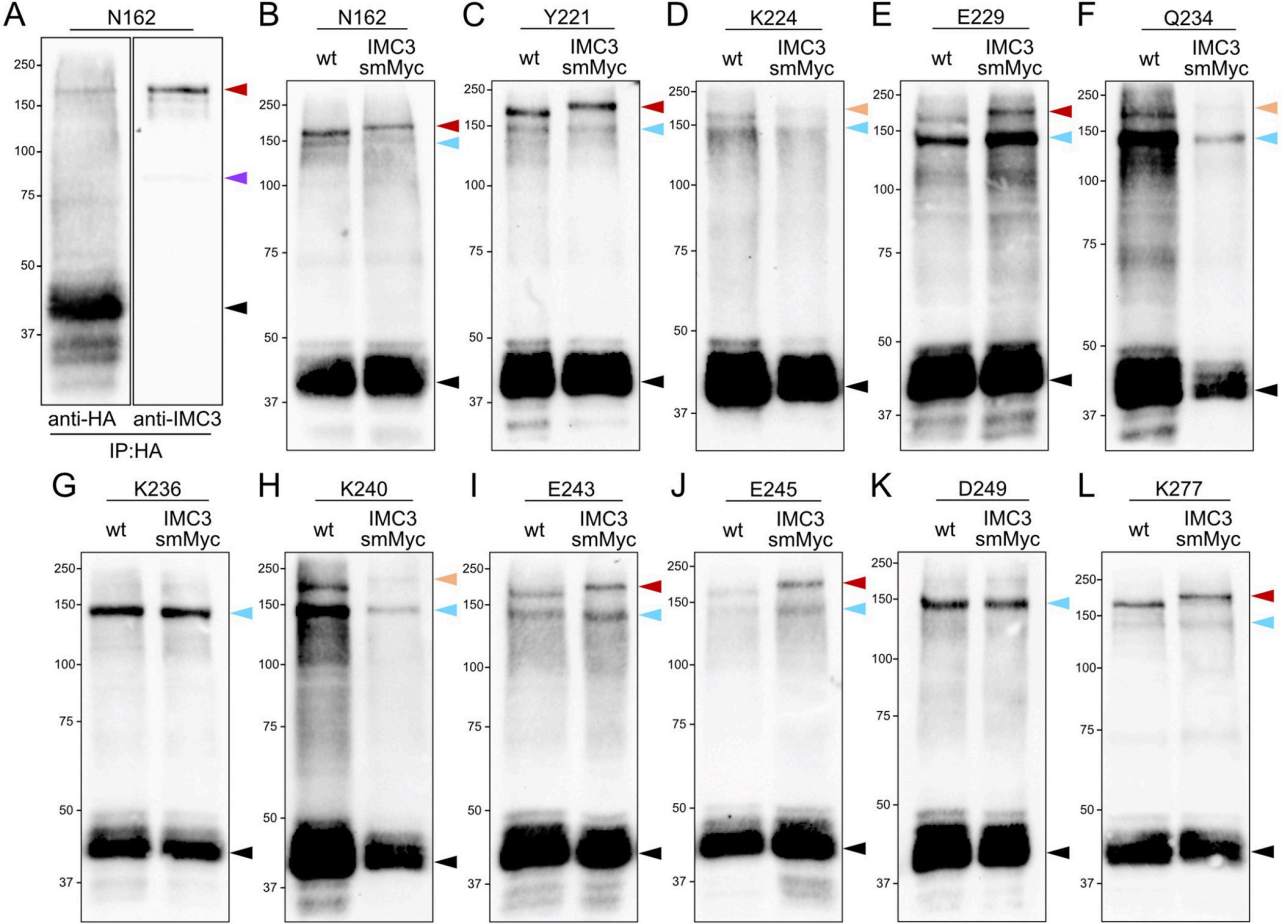
IMC3 binds the IMC6 alveolin domain at multiple sites. (**A**) Western blot of IMC6-N162* denaturing IP following Azi incorporation and UV treatment. Black arrowhead represents uncrosslinked IMC6^2-290^-3xHA (about 45 kDa). Purple arrowhead represents uncrosslinked IMC3 nonspecifically captured in the IP (about 85 kDa). Red arrowhead represents the crosslink-of-interest, seen in both the HA and IMC3 blots (about 175 kDa). (**B**-**L**) Western blot of whole cell lysates following Azi incorporation and UV treatment. For each residue, both WT and IMC3-smMyc strains were subjected to photoreactive crosslinking and analyzed on the same blot for direct comparison. Black arrowheads represent uncrosslinked IMC6^2-290^-3xHA (about 45 kDa). Blue arrowheads indicate the crosslinked product at about 140 kDa, which is not shifted further by the IMC3-smMyc tag. Red arrowheads represent the crosslinks-of-interest, which migrate even higher in the IMC3-smMyc sample (about 175 kDa in the WT vs. 200 kDa in the IMC3-smMyc). Orange arrowheads represent faint crosslinks-of-interest with similarly higher migration in the IMC3-smMyc sample. Every blot was detected with anti-HA. Azi, *p*-azidophenylalanine; HA, hemagglutinin; IP, immunoprecipitation; smMyc, spaghetti monster Myc; WT, wild-type.

To identify the crosslinked partners of IMC6, we considered candidates whose localization resembles IMC6 and predicted molecular weight represents the upshifted product. For the larger crosslinked products at 140 and 175 kDa, IMC3 appeared to be the most likely candidate. To determine if IMC3 is a crosslinked partner of IMC6, we chose N162 to carry out preliminary experiments as proof of concept. We first performed a denaturing co-IP experiment, in which the irradiated and crosslinked samples are boiled in 1% SDS before being diluted to standard RIPA buffer conditions for the IP (Fig 8A) [22]. Probing with anti-hemagglutinin (HA) indicated that we successfully purified both uncrosslinked and crosslinked products, migrating at the same molecular weights as the whole cell lysate samples in Fig 7B. Probing with anti-IMC3 demonstrated that IMC3 is indeed the binding partner for IMC6 at this residue. As a complementary approach, we endogenously tagged IMC3 with a spaghetti monster Myc (smMyc) epitope tag in the amber mutant parasite strain, performed photoreactive crosslinking, and compared the upshift sizes between the untagged and smMyc-tagged samples (Fig 8B). We observed a significant size difference between the untagged and tagged versions, confirming that IMC3 binds IMC6 at residue N162. Based on this preliminary evidence, we evaluated the remaining residues using the endogenous tagging approach as this provides a definitive identity of the binding partner and produces more consistent results than co-IPs.

We thus endogenously tagged IMC3 with smMyc in the other 10 residues of similar-sized crosslinked products, performed photoreactive crosslinking on both the untagged and tagged versions, and analyzed each pair by western blot (Fig 8C–8L). Five of these residues (Y221, E229, E243, E245, and K277) showed an unmistakable size difference due to the smMyc tag, from approximately 175 kDa in the untagged version to about 200 kDa in the tagged version (indicated by red arrowheads). Three other residues (K224, Q234, and K240) showed a similar size difference (orange arrowheads). While these almost certainly bind to IMC3 as well, we denoted them as *likely* IMC3-binding due to the fainter upshifts of the tagged versions. Nonetheless, this demonstrates that IMC3 binds IMC6 via an array of residues spanning the alveolin domain.

We also observed upshifts migrating at about 140 kDa throughout the alveolin domain (blue arrowheads). These, however, did not shift higher in the IMC3^smMyc^ strain, indicating that IMC3 is not the binding partner for this crosslinked product. While the 140 kDa crosslink was the only product for K236 and D249, it was an additional product for all other residues. This suggests that the same residue may have the capacity to interact with both IMC3 and a second, unknown protein or that some background occurs with this shifted product.

### ILP1 is another binding partner of IMC6

**Fig 9:**
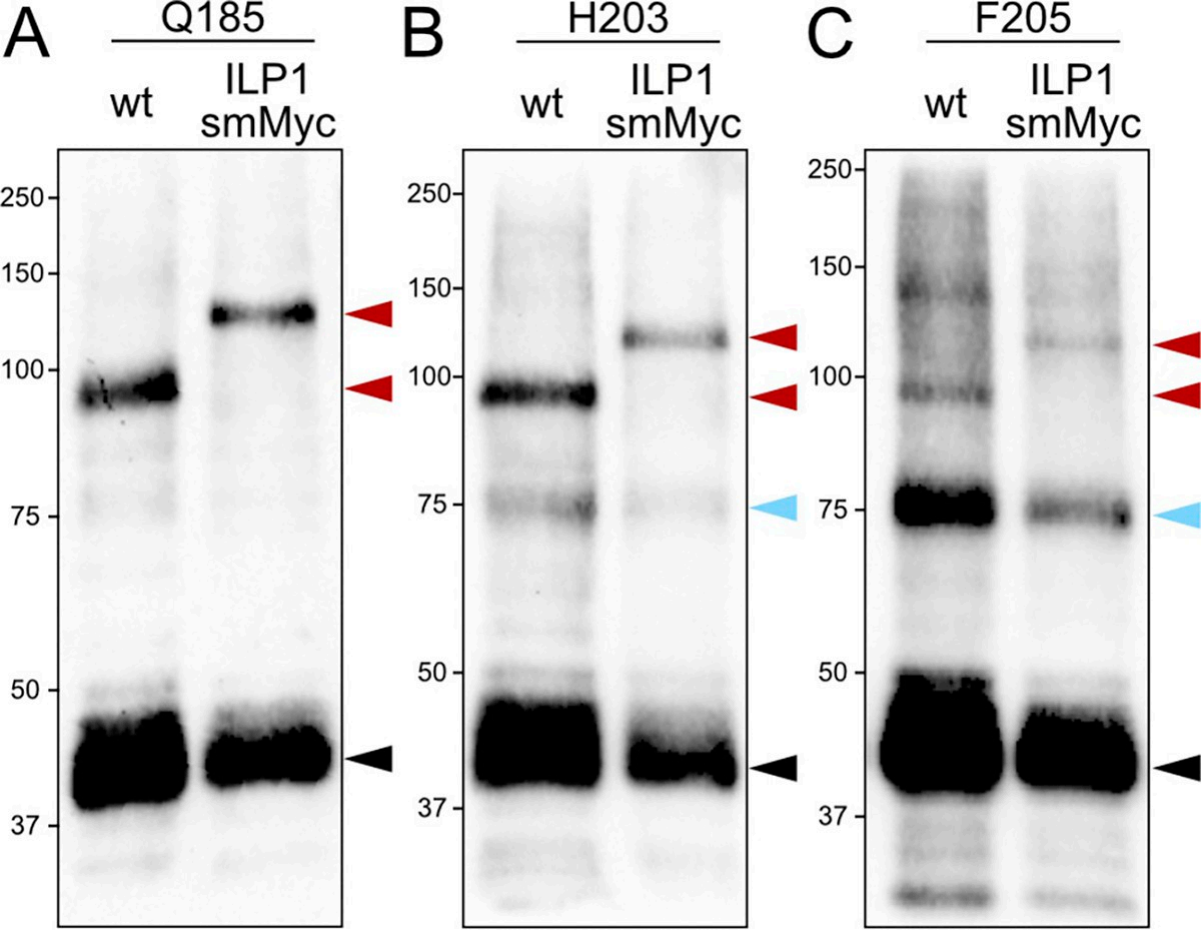
ILP1 binds IMC6 in the alveolin domain. (**A**-**C**) Western blot of whole cell lysates of the indicated residues following Azi incorporation and UV treatment. For each residue, both wild-type and ILP1-smMyc strains were subjected to photoreactive crosslinking and analyzed on the same blot for direct comparison. Black arrowheads represent uncrosslinked IMC6^2-290^-3xHA (about 45 kDa). Blue arrowheads indicate crosslinked products at about 75 kDa, which is not shifted further by the ILP1-smMyc tag. Red arrowheads represent the crosslinks-of-interest, which migrate even higher in the ILP1-smMyc sample (approximately 95 kDa vs. approximately 120 kDa). Every blot was detected with anti-HA. Azi, *p*-azidophenylalanine; HA, hemagglutinin; ILP1, IMC localizing protein 1; smMyc, spaghetti monster Myc.

We next explored the other 3 residues with crosslinked upshifts at about 75 and 95 kDa. While the Q185 crosslinked product migrated prominently at approximately 95 kDa, the H203 and F205 crosslinked products migrated at both 75 and 95 kDa, though with opposite intensities (Fig 7A and 7B). We used a similar criteria of protein localization and predicted molecular weight to select IMC localizing protein 1 (ILP1) as the prime candidate for these upshifts. Employing the same approach, we endogenously tagged ILP1 with smMyc in each amber mutant parasite strain and performed photoreactive crosslinking on both untagged and tagged strains (Fig 9A–9C). Upon analysis by western blot, we observed a dramatic difference in the migration size between the untagged (about 95 kDa) and smMyc-tagged (about 120 kDa) versions for all 3 residues. This confirms that ILP1 is indeed the binding partner for IMC6 at these residues. Although the H203 and F205 crosslinked products migrated at 2 different sizes, only the higher 95 kDa was confirmed to bind ILP1 while the target for the lower 75 kDa crosslink remains unknown. This indicates that H203 and F205 may bind a second protein in addition to ILP1 or that this product represents background. These confirmed products demonstrate that IMC6 directly binds another cytoskeletal protein via the alveolin domain, underscoring the highly connected nature of the SPN.

## Discussion

By exploring the function and interactions of IMC6, we reveal important insights into the apicomplexan SPN with potentially broader implications for other alveolates. Of the 5 alveolin proteins that are predicted to be highly fitness-conferring (IMC1, IMC3, IMC4, IMC6, and IMC10), only IMC10 had been examined prior to this study. Conditionally knocking down IMC10 attributed one of the roles of the SPN as a membrane-tether for the mitochondria [35]. Interestingly, however, the knockdown did not affect parasite fitness, though this may be due to an incomplete knockdown of the conditional system. Other alveolins that have been functionally characterized include IMC7, IMC12, and IMC14, which were shown to have modest involvement in tensile strength of extracellular parasites but no growth defects [24]. In addition, IMC15 was shown to restrict the number of daughter buds per round of division, but again, no growth defects were reported upon its disruption [24]. Thus, this study represents one of the first major perturbations of the *T*. *gondii* SPN, revealing that it is a critical structure that provides the parasite its elongated shape as well as a scaffold for new daughter cells.

Moreover, we clearly link the parasite cytoskeleton to its shape, motility, and invasion. This phenomenon has been reported several times in the past. For example, disrupting TgPhIL1 resulted in shorter and wider parasites with significant motility defects but surprisingly no invasion defects [30,36]. On the other hand, knocking out TgCBAP/TgSIP1 yielded parasites with less severe shape defects but did cause motility and invasion defects [37,38]. Knocking down GAPM1a caused the most severe defects in motility with a link to microtubule morphology and stability but surprisingly minor impact on parasite shape [39]. We show here that the IMC6 knockout resulted in almost completely rounded parasites, demonstrating that the integrity of the SPN is a key determinant for maintaining parasite shape. We were surprised, though, that this extreme morphological defect did not cause a more severe phenotype in motility and invasion, as both processes were only modestly affected. As microneme secretion remains intact in the knockout parasites, our data suggest that exocytosis, rather than physical shape, is more important for motility. For delayed invasion, one likely explanation is that swollen parasites are slower to reorient their apical end towards the host cell to engage the invasion machinery. It is also possible that these subtle defects in vitro may be more dramatic in vivo, but this requires further study.

Beyond *T*. *gondii*, some of the *Plasmodium berghei* alveolins have been shown to be involved in maintaining parasite shape and motility in sporozoites and ookinetes, broadening the significance of the SPN to more distant apicomplexan organisms [25,40–42]. A recent study corroborated this finding in the human malarial parasite *Plasmodium falciparum*, demonstrating that the SPN provides similar structural functions during the asexual blood stages [43]. The SPN also harbors cytoskeletal proteins that do not possess alveolin domains, one of which is TgILP1 and the orthologous PbG2. Both were shown to be essential in their respective organisms [19,20,44], again highlighting the conserved functions of these cytoskeletal proteins and the SPN as a whole. One major difference between these parasites is the expanded number of cytoskeletal IMC proteins in *T*. *gondii*. While it is likely that additional *Plasmodium* IMC proteins will be discovered in the future, the expanded proteome of the *T*. *gondii* IMC exhibits important differences in protein composition. Investigation of additional TgIMC proteins may provide further insights into the divergence of these organisms.

**Fig 10:**
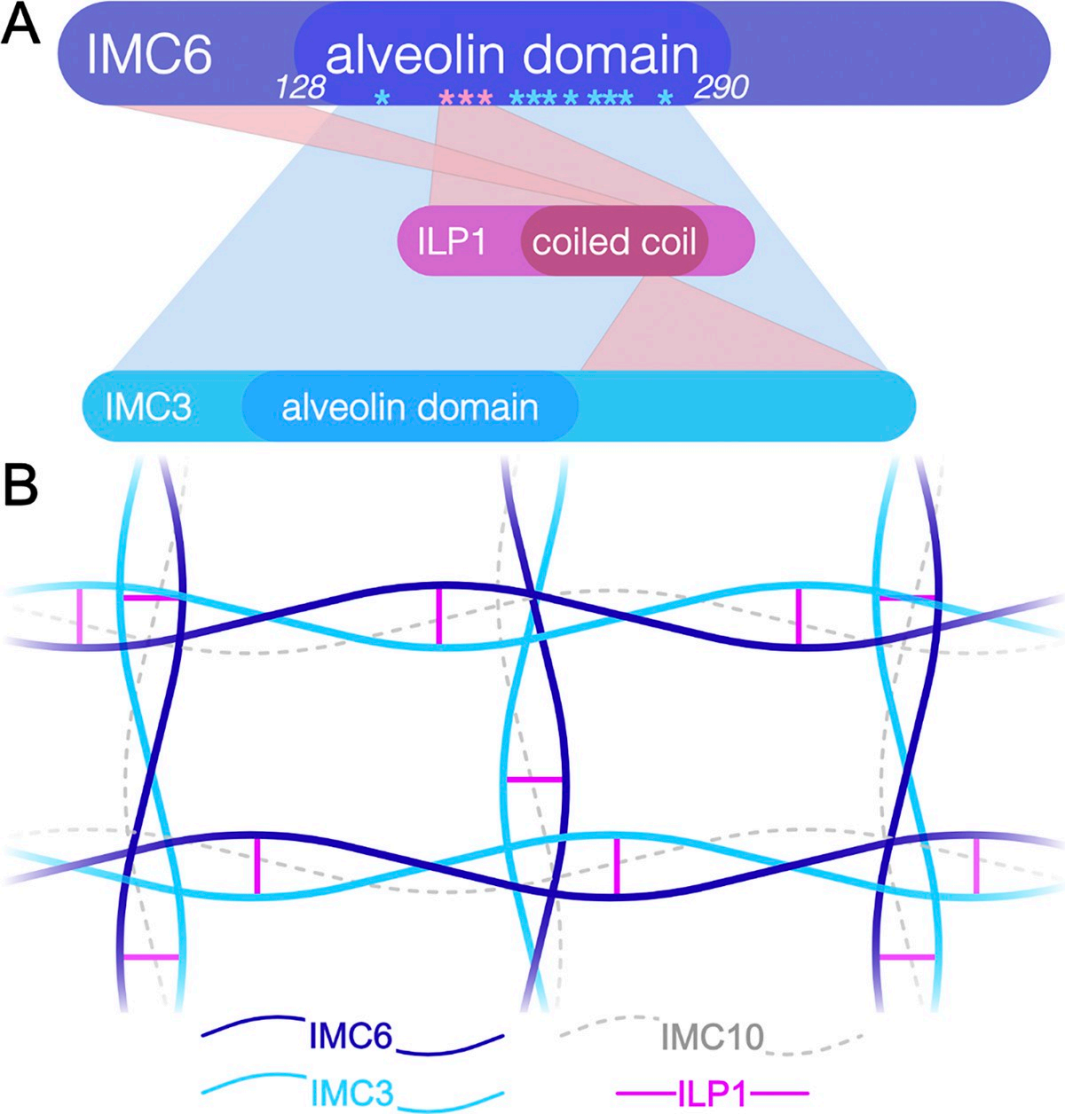
Proposed model of alveolin interactions. (**A**) The IMC6 alveolin domain mediates binding to IMC3 and ILP1. Blue and red shades represent putative interactions with IMC3 and ILP1, although the precise binding interfaces on these interactors are unknown. Asterisks indicate residues in the IMC6 alveolin domain that are verified to interact with IMC3 or ILP1. The ILP1 coiled coil domain was previously shown to interact with IMC6 and IMC3 [22]. (**B**) Proposed diagram of a lattice of alveolin filaments made by IMC6, IMC3, and IMC10. IMC10 is included as a likely interactor due to its daughter-enriched localization. ILP1 is depicted as a reinforcing connection between IMC6 and IMC3, though it is possible that ILP1 is part of the major filament instead.

Historically, alveolin domains have been thought to mediate the formation of intermediate filament-like structures that constitute the SPN. Evidence for this includes their detergent-resistant biochemical properties and the highly conserved repeat motifs that suggest structural functions [1,15]. Moreover, domain analyses demonstrated that the alveolin domain is sufficient for TgIMC3 localization and partially sufficient for TgIMC8 [18]. In other apicomplexans, only sparse evidence exists. The alveolin domain of PbIMC1h was shown to be required for the protein’s localization, and expressing a synthetic protein with alveolin-like motifs in *Tetrahymena* was remarkably sufficient for associating with cytoskeletal structures [25,45]. Here, we demonstrate that the IMC6 alveolin domain binds directly to another alveolin, IMC3, using multiple contact sites (Fig 10). These sites of interaction span almost the entirety of the alveolin domain suggesting the 2 proteins are highly interwoven. This extensive binding likely increases the strength and avidity of their interaction, possibly leading to the high tensile strength of filaments. While this method only maps binding interfaces on the bait protein, it is likely that similar experiments using IMC3 would also show this highly interconnected pattern, as the IMC3 alveolin domain is also sufficient for trafficking to the SPN [18].

We also demonstrate that ILP1 binds to the alveolin domain of IMC6 (Fig 10). Unlike IMC3, however, ILP1 only binds to IMC6 in a 20-residue region of the alveolin domain (aa185-205). In addition, we have previously shown that the variable N-terminal region of IMC6 is required for crosslinking to ILP1 [22]. It is possible that ILP1 binds to both regions of IMC6 or that the N terminus indirectly facilitates ILP1 binding to the alveolin domain. This region also contains 2 predicted palmitoylated residues (C89 and C90) that may help IMC6 tether to the IMC membranes and optimally incorporate into the SPN [46]. Regardless, this study confirms that ILP1 forms tight interactions with alveolin proteins, highlighting the importance of alveolin-associated proteins like ILP1 as they are also critical for the organization and function of the SPN. As IMC6, IMC3, and ILP1 share an identical localization pattern with basal levels in the maternal IMC and enrichment in the daughter IMC, they appear to form an essential subgroup of “daughter-enriched” protein complexes. IMC10 also shares this localization pattern but we have yet to determine how it links to the other 3. Another likely pairing of alveolins are IMC1 and IMC4, which are distributed more equally between the maternal and daughter IMC. Investigating the interactions of this pair using the UAA system may reveal a distinct subcomplex. Interestingly, proteolytic cleavage of IMC1 was shown to mark the transition of the detergent-labile SPN of daughter cells to the detergent-resistant SPN of maternal cells [47]. While IMC3/6/10 and ILP1 are also present in both daughter and maternal SPNs, how they contribute to the maturing process remains unknown. Together, our results corroborate the long-held hypothesis that the alveolin domains are key mediators of SPN architecture and provide a glimpse into the binding interfaces that link these filaments to one another. It will be interesting to dissect which interactions are essential for SPN integrity and to compare our results to future crystallographic or cryoET studies that promise to reveal the entire organization of the network.

## Materials and methods

### Ethics statement

Our protocol was approved by the UCLA Institutional Animal Care and Use Committee (Chancellor’s Animal Research Committee protocol: 2004–005). Mice were euthanized when the animals reached a moribund state, and euthanasia was performed following AVMA guidelines.

### *T*. *gondii* culture

Parental *T*. *gondii* RHΔ*hxgprt*Δ*ku80* (wild-type) and subsequent strains were grown on confluent monolayers of human foreskin fibroblasts (BJ, ATCC, Manassas, VA) at 37°C and 5% CO_2_ in Dulbecco’s Modified Eagle Medium (DMEM) supplemented with 5% fetal bovine serum (Gibco), 5% Cosmic calf serum (Hyclone), and 1x penicillin-streptomycin-L-glutamine (Gibco). For the motility experiments, HFFs and parasites were cultured in 10% v/v heat-inactivated FBS. Drug selections were performed using 1 μM pyrimethamine (dihydrofolate reductase-thymidylate synthase [DHFR-TS]), 50 μg/mL mycophenolic acid-xanthine (HXGPRT), or 40 μM chloramphenicol (CAT) [48–50]. Homologous recombination to the UPRT locus was negatively selected using 5 μM 5-fluorodeoxyuridine (FUDR) [51].

### PCR and plasmid construction

#### Knockout and complementation of *IMC6*

To knock out *IMC6*, the protospacer was designed to target the coding region of IMC6 (TGGT1_220270) and ligated into the pU6-Universal plasmid using P1-2 [52]. The homology-directed repair (HDR) template included 40 bp of homology immediately upstream of the start codon and 40 bp of homology approximately 200 bp downstream of the stop codon (P3-4). The HDR template was PCR amplified from a pJET vector containing the HXGPRT selectable marker driven by the NcGRA7 promoter. For transfection, approximately 50 μg of the gRNA-carrying pU6-Universal plasmid was precipitated in ethanol, and the PCR-amplified HDR template was purified by phenol-chloroform extraction and precipitated in ethanol. Both constructs were electroporated into the RHΔ*hxgprt*Δ*ku80* (wild-type) parasite strain. Transfected cells were allowed to invade a confluent monolayer of HFFs overnight, and appropriate selection was subsequently applied. Successful knockout was confirmed by IFA, and clonal lines were obtained through limiting dilution.

All IMC6 complementation constructs were modified from those used in [22]. Each construct contains either the full-length or truncated version of the IMC6 coding sequence, a C-terminal V5 tag, and UPRT homology regions to drive this cassette into the *UPRT* locus. For this study, the ILP1 promoter was replaced with the IMC6 promoter in each plasmid using Gibson assembly. The IMC6 promoter was amplified from genomic DNA (P5-6). The full-length IMC6 coding sequence along with the rest of the plasmid was amplified using P7/P9. These amplicons were purified and ligated using the NEBuilder HiFi DNA Assembly kit to generate the final plasmid. The same process was followed for each truncation: IMC6^2-290^ was amplified using P7/P9, IMC6^128-290^ was amplified using P8/P9, and IMC6^128-444^ was amplified using P8/P9. About 100 μg of each complementation plasmid was linearized with DraIII-HF and transfected into Δ*imc6* parasites along with a pU6-gRNA that targets the UPRT coding region. Selection was performed with FUDR for replacement of *UPRT* [51], and potential clones were screened by IFA.

#### Amber stop codon mutagenesis

The plasmid used to express the amber stop codon tRNA and its orthogonal tRNA synthetase is described in [22]. To generate amber mutants, we first created the IMC6 expression construct, which contains the IMC10 promoter driving the coding sequence of IMC6^2-290^ with a C-terminal 3xHA epitope tag as well as the DHFR-TS selectable marker. We then used the NEB Q5 mutagenesis kit to exchange the corresponding residue with the amber stop codon TAG using primers P10-P85. About 100 μg of each amber mutant plasmid was linearized with DraIII-HF, transfected into wild-type parasites stably expressing pGra-E2AziTy.HPT.3xtRNA, and selected with pyrimethamine.

#### Endogenous epitope tagging

All genes were C-terminally tagged in this study. A pU6-Universal plasmid was generated containing a protospacer against the 3′ UTR of the target protein approximately 100 bp downstream of the stop codon. The HDR template was PCR amplified using the Δ*ku80*-dependent LIC vector pSmMyc.LIC-CAT, which includes the epitope tag, 3′ UTR, and the selection cassette. The 60-bp primers include 40 bp of homology immediately upstream of the stop codon or 40 bp of homology within the 3′ UTR downstream of the CRISPR/Cas9 cut site. Primers P86-113 were used to generate each of these constructs.

### Antibodies

The HA epitope was detected with mouse monoclonal antibody (mAb) HA.11 (BioLegend; 901515) or rabbit polyclonal antibody (pAb) anti-HA (Invitrogen; PI715500). The Ty1 epitope was detected with mouse mAb BB2 [53]. The V5 epitope was detected with mouse mAb anti-V5 (Invitrogen; R96025). *Toxoplasma*-specific antibodies include mAb mouse anti-IMC1 [54], pAb rabbit anti-IMC3 [16], pAb rabbit anti-IMC6 [22], pAb rat anti-IMC10 [35], pAb rabbit anti-IMC12 [55], mouse mAb anti-ISP1 [56], mAb mouse anti-MIC2 [57], mAb mouse anti-ROP7 [58], pAb rabbit anti-ROP13 [59], pAb rat anti-GRA39 [60], mAb mouse anti-F_1_β subunit (5F4) [61], mAb mouse anti-ATrx1 (11G8) [62], pAb guinea pig anti-NHE3 [63], and mAb mouse anti-SAG1 (DG52) [64].

### Immunofluorescence assay and western blot

For IFAs, HFF cells were grown to confluence on glass coverslips. Around 18 to 36 hpi with *T*. *gondii*, the coverslips were fixed with 3.7% formaldehyde in PBS and processed for immunofluorescence as described [59]. Primary antibodies were typically incubated for 1 hour, washed with PBS 3 to 5 times, species-specific secondary antibodies (Alexa Fluor 488/594) were incubated for 1 hour, and washed with PBS at least 3 to 5 times. Coverslips were then mounted in Vectashield (Vector Labs, Burlingame, CA), viewed with an Axio Imager.Z1 fluorescent microscope (Zeiss), and processed with ZEN 2.3 software (Zeiss).

For western blot, whole cell parasite lysates were prepared in 1x Laemmli sample buffer with 100 mM DTT and boiled at 100°C for 10 minutes. Lysates were resolved by SDS-PAGE and transferred to nitrocellulose membranes. Membranes were incubated in primary antibodies for 1 hour, washed in PBS with 0.01% TWEEN-20, incubated in secondary antibodies conjugated to horseradish peroxidase for 1 hour, and washed with PBS-TWEEN. Chemiluminescence was induced using the SuperSignal West Pico substrate (Pierce) and imaged on a ChemiDoc XRS+ (Bio-Rad, Hercules, CA).

### Detergent extraction and transmission electron microscopy

An on-grid extraction method was applied to make parasite ghosts. First, 4 μl of the parasite resuspension was loaded on a glow-discharged grid with continuous carbon film. After settling for 2 minutes, the extra buffer was blotted away from the side of the grid, and the grid was transferred upside down onto the top of a 20 μl drop of detergent extraction buffer (1% Triton X-100, 0.5% DOC, 50 mM Tris, 150 mM NaCl). The grid was left to float on top of the drop for 5 minutes to lyse the parasites. The grid was then blotted to remove excess buffer and transferred to a second drop of detergent extraction buffer for 5 minutes. This process was repeated 6 times and then washed 4 times in a similar manner using 20 μl of PBS. The grid was finally stained with 2% uranyl acetate. The negatively stained grid was imaged on FEI Tecnai T12 equipped with a CCD camera, operated at 120 kV.

### Parasite functional assays

#### Plaque assay

HFF monolayers were grown in 6-well plates to confluence and subsequently infected with 200 parasites/well and allowed to form plaques for 7 days. Cells were then fixed with ice-cold methanol and stained with crystal violet. The areas of 30 plaques/well were measured using ZEN software (Zeiss) and Fiji. Plaque efficiency was measured by counting the number of plaques/well divided by the number of parasites inoculated/well. Triplicate experiments were conducted for all plaque assays. Graphical and statistical analyses were performed using Prism GraphPad 8.0. Significance of plaque areas was determined by two-way ANOVA, and significance of plaque efficiency was determined by multiple two-tailed *t* tests.

#### Invasion assay

Invasion assays were modified from previous protocols [65]. Briefly, parasites were mechanically released through a syringe, resuspended in Endo buffer, and settled onto coverslips with nonconfluent HFF monolayers for 20 minutes. Endo buffer was then replaced with warm D1 media (DMEM, 20 mM HEPES, 1% fetal bovine serum) and incubated at 37°C for the appropriate invasion time points (5 minutes, 20 minutes, 60 minutes). Coverslips were then fixed and blocked (3% BSA in PBS), and extracellular parasites were stained with anti-SAG1 antibodies. Coverslips were then permeabilized (3% BSA, 0.2% Triton X-100 in PBS), and all parasites were stained with anti-F_1_β ATPase antibody and, subsequently, the secondary antibodies. Host nuclei were stained with Hoechst during the final rounds of PBS washing. Parasites were scored as invaded (SAG1−, F_1_β+) or not (SAG1+, F_1_β+) per host nucleus. These assays were performed in triplicate with at least 25 host nuclei across 7 fields for each replicate. Significance was determined using two-way ANOVA.

#### Egress assay

Parasites were grown on a monolayer on coverslips until most vacuoles contained 16 or 32 parasites. Coverslips were washed twice with prewarmed PBS and incubated with A23187 (or DMSO control) diluted in PBS at 37°C for 4 minutes. Coverslips were then fixed and stained with rabbit anti-IMC12 antibodies. At least 15 fields of about 10 vacuoles per field were counted for 3 replicate coverslips for each condition.

#### Microneme secretion assay

Microneme secretion assays were performed as previously described [12,66]. Briefly, parasites were grown for 30 hours (or about 40 hours for knockout strain), and intracellular parasites were collected by mechanical release through a 27-gauge needle. After washing twice in D1 media, parasites were resuspended in prewarmed D1 media containing 1 μM A23187 (or DMSO control) for 4 minutes at 37°C. Secretion was arrested by cooling on ice, and parasites were pelleted at 1,000 × *g* for 5 minutes at 4°C. The supernatant was collected and centrifuged again at 1,000 × *g*. The resulting supernatant was mixed with 5x sample buffer and assessed by SDS/PAGE and western blot analysis.

#### Replication defects by IFA

Confluent HFF monolayers on glass coverslips were infected at low MOI, incubated for 1 hour at 37°C, and extracellular parasites were washed away by media changes. Then, 24 and 32 hpi, coverslips were fixed with 3.7% PFA, processed for immunofluorescence, and labeled with anti-ISP1 and anti-IMC3. To score normal versus abnormal vacuoles, >300 vacuoles across at least 15 fields were counted. Vacuoles were categorized as abnormal if any one of the replication defects shown in Fig 4 were present in a vacuole. All IFAs were performed in triplicate, and significance was determined using multiple two-tailed *t* tests.

To stain extracellular parasites, wild-type, Δ*imc6*, and IMC6c strains were allowed to egress naturally, collected, and washed in PBS before being settled onto coverslips coated with poly-L-lysine (Sigma Aldrich). Standard IFA procedure was followed to stain samples with mouse anti-ISP1 and rabbit anti-IMC3, making sure to be extra gentle with all wash steps to avoid disturbing the settled parasites. Quantifications were performed by counting >300 parasites across at least 15 fields. Three replicates were conducted, and significance was determined by multiple two-tailed *t* tests.

### ImageStream flow cytometry

Freshly lysed (or mechanically released) extracellular parasites were processed for immunofluorescence in solution. Parasites were fixed with 3.7% formaldehyde, permeabilized, blocked, and stained with mouse anti-IMC1 followed by anti-mouse Alexa Fluor 488. For each parasite strain, at least 20,000 individual images were acquired using the INSPIRE acquisition software of the ImageStream MKII (Cytek Biosciences, Seattle, WA) imaging flow cytometer with 60× magnification and a core width of 6 μM. The raw images can be found in 10.5281/zenodo.13333981, and the gating strategy used to obtain single cells can be found in S1 Data.

All analysis including gating was performed using IDEAS software v6.3.26 (Cytek Biosciences, California). The Circularity feature measures the radial variance from the central point of the object. Higher values equate to low radial variance and indicate a highly circular object, while lower values indicate that the object has a high amount of radial variance and have a less circular morphology. The Elongated ML classifier attempts to identify objects that have an elongated shape and assigns a value to each object, indicating confidence. The more positive the value is, the more likely the object is to be elongated. Negative values represent poor prediction confidence, and it is more likely that these objects are circular rather than elongated.

### 3D motility assay

Parasites were mechanically released by syringe lysis and filtered through a 3-μm Whatman Nucleopore filter (Milipore Sigma). Parasites were then centrifuged and resuspended at 5 × 10^8^ parasites/mL in live cell imaging solution (LCIS) (155 mM NaCl, 3 mM KCl, 2 mM CaCl_2_, 1 mM MgCl_2_, 3 mM NaH_2_PO_4_, 10 mM HEPES, and 20 mM glucose (Sigma-Aldrich) with 20 μg/mL Hoechst 33342 to visualize the nuclei. Gently, 1:3:3 volumes of parasites:LCIS:Matrigel were mixed, in that order, and pipetted into a Pitta chamber [30]. Flow cells were incubated for 7 minutes at 27°C and then 3 minutes at 35°C before imaging. Images were captured using a Nikon Eclipse TE300 epifluorescence microscope (Nikon Instruments, Melville, NY) equipped with a 20X Plan Apo λ objective and a NanoScanZ piezo Z stage insert (Prior Scientific, Rockalnd, MA). Fluorescence images with 1,024 pixel × 384 pixel were captured with an iXON Life 888 EMCCD camera (Andor Technology) driven by NIS Elements software v.5.11 (Nikon Instruments). Fluorescence excitation was controlled using a pE-4000 LED illumination system (CoolLED, Andover England). Images were captured for 80 seconds. The camera was set to trigger mode, no binning, readout speed of 30 Mhz, conversion gain of 3.8×, and an EM gain of 300. Image stacks consisted of 41 x-y slices captured 1 μm apart in z for 16 ms. Brightfield images with 1,024 × 1,024 pixel were captured over 5 minutes using a 60X Plan Apo λ objective. Brightfield image stacks consisted of 21 x-y slices captured 1 μm apart in z for 40 ms.

Trajectory data were analyzed with Imaris ×64 v. 9.9.0 software (Bitplane AG, Zurich, Switzerland). Parasites were tracked using an estimated spot volume of 3.0 × 3.0 × 6.0 μm. Tracks were gated for 10-second duration and 2.8 μm track displacement in order to be considered actively moving. The 2.8-μm minimum displacement parameter was determined by both heat-killing and treating all parasite strains with cytochalasin D (CD). *T*. *gondii* tachyzoites were heat-killed by incubating at 56°C for 30 minutes. For CD treatment, parasites were incubated in LCIS containing 1 μM CD and 20 μg/mL Hoechst 33342 for a minimum of 20 minutes and maintaining a concentration of 1 μM CD within the flow cells. All 3D trajectory data were acquired from 3 biological replicates, each consisting of 3 technical replicates.

### UAA photoreactive crosslinking

#### Rationale for choosing amino acids to crosslink

We first used JPred4 to predict secondary structures, which indicated that the alveolin domain largely consists of beta-sheets, as expected [33]. While the short beta-sheets of 1 to 2 amino acids are likely not real, they were included in Fig 6 as predicted to minimize bias. To prioritize amino acids, we considered 2 factors. First, our previous experience with photoreactive crosslinking showed that charged residues tend to perform better in photoreactive crosslinking [22]. It is unclear whether this is a bias of the protocol or a biologically relevant pattern of IMC-associated cytoskeletal proteins. Second, Nath Jha and colleagues showed that V, L, I, A, T, and F are bioinformatically more likely to make contact sites on beta-sheets [34].

#### Whole cell lysates

All parasites used for photoreactive crosslinking stably express the synthetase/tRNA cassette and IMC6^2-290^ with the appropriate amber mutation. These parasites were allowed to infect confluent HFFs for 16 to 24 hours, after which the media was replaced with complete DMEM supplemented with 1 mM Azi. After 24 hours of incubation in Azi media, freshly lysed (or syringe-lysed) parasites were collected, resuspended in 2 ml PBS, and deposited into 6-well plates. The plates were floated on an iced water slurry without lids and irradiated for 20 minutes in a Spectrolinker XL-1000 UV crosslinker with 365-nm (UV-A) bulbs. Cells were collected by centrifugation and lysed directly in 80 to 200 μl of sample buffer for SDS-PAGE and subsequent western blot. For quantification, the band intensities of each primary crosslinked product and each uncrosslinked IMC6^2-290^ were measured using ImageLab software (BioRad). These values were then normalized to background intensities. The ratio was determined by dividing the crosslinked product by the corresponding uncrosslinked product for each applicable lane.

#### Denaturing co-IP

This protocol was followed very closely as previously described [22]. Briefly, following irradiation, parasites were lysed in an extremely harsh SDS buffer (1% SDS, 150 mM NaCl, 50 mM Tris (pH 8.0)) and boiled at 100°C for 10 minutes. This lysate was then centrifuged, and the supernatant was diluted 10-fold to RIPA conditions (50 mM Tris, 150 mM NaCl, 0.1% SDS, 0.5% NP-40, 0.5% DOC). IP was then conducted using anti-HA agarose beads (Roche) overnight at 4°C. Affinity captured proteins were eluted directly in sample buffer, agarose beads removed by centrifugation, boiled for 10 minutes, and analyzed on SDS-PAGE.

### Mouse virulence assays

Large vacuoles of RHΔ*hxgprt* (wild-type), Δ*imc6*, and IMC6c parasites were syringe-lysed and resuspended in Opti-MEM medium (Thermo Fisher Scientific) prior to intraperitoneal injection into female C57BL/6 mice (4 mice per parasite strain) at the appropriate dosages. An aliquot of this parasite resuspension was used for plaque assays to assess the viability of each injected parasite strain. Mice were monitored for symptoms of infection, weight loss, and survival for 30 days. Survival graphs were generated on Prism GraphPad 8.0.

## Supporting information

S1 Fig Features used in the ML analysis. Table of features used in the Elongated ML classifier and their weight.
(TIFF)

S2 Fig Additional TEM of wild-type and knockout parasites. Detergent extracted and negatively stained parasites, highlighting the microtubules and subpellicular network.
(TIFF)

S3 Fig Microneme secretion and induced egress is unaffected in Δ*imc6* parasites. (**A**) Western blot of secreted proteins from WT and Δ*imc6* parasites. The calcium ionophore A23187 was used to induce microneme secretion. MIC2 was detected with anti-MIC2, and the constitutively secreted dense granule protein GRA39 was used as a control, detected with anti-GRA39. (**B**) Calcium ionophore-induced egress assay, quantified by counting the number of vacuoles that have egressed or not. Data are plotted as the mean ± SD, and significance was calculated using two-tailed *t* tests. The raw data underlying this figure can be found in S1 Data.
(TIFF)

S4 Fig The apicoplast, mitochondria, PLV/VAC, micronemes, and rhoptries are unaffected in Δ*imc6* parasites. (**A**-**E**) IFAs of mature and budding wild-type or Δ*imc6* parasites, showing typical morphology of the indicated organelles. (**A**) The apicoplast was detected with anti-ATrx1 (magenta). (**B**) Mitochondria were detected with anti-F_1_β (magenta). (**C**) The PLV/VAC was detected with anti-NHE3 (magenta). (**D**) Micronemes were detected with anti-MIC2 (magenta). (**D**) Rhoptries were detected with anti-ROP7 (magenta). All IFAs were costained with anti-IMC3 (green). All scale bars are 2 μm.
(TIFF)

S5 Fig The basal complex appears intact in Δ*imc6* parasites. (**A**) IFAs of wild-type parasites show proper BCC1 localization. (**B**) IFAs of knockout parasites show proper BCC1 localization despite morphological and replication defects. White arrows point to maternal basal complexes and orange arrows point to daughter basal complexes. (**C**) IFAs of incompletely separated knockout parasites, which still show proper BCC1 localization. White arrows point to maternal basal complexes and orange arrows point to daughter basal complexes. BCC1 was detected with mouse anti-HA. All scale bars are 2 μm.
(TIFF)

S6 Fig Representative extracellular images of complemented strains. Phase contrast images of extracellular parasites show partial rescue of the shape defect. All scale bars are 5 μm.
(TIFF)

S1 Video Representative field of WT 3D motility. (MP4)

S2 Video Close-up view of typical WT 3D motility. (MP4)

S3 Video Representative field of Δ*imc6* 3D motility. (MP4)

S4 Video Close-up view of abnormal Δ*imc6* trajectory 1. (MP4)

S5 Video Close-up view of abnormal Δ*imc6* trajectory 2. (MP4)

S6 Video Close-up view of abnormal Δ*imc6* trajectory 3. (MP4)

S7 Video Representative field of IMC6c 3D motility. (MP4)

S8 Video Close-up view of restored IMC6c 3D motility. (MP4)

S1 Table Ratio of uncrosslinked to crosslinked products. Residues chosen for partner identification are bolded.
(XLSX)

S2 Table Oligonucleotides used in this study. All primers are in 5′ to 3′ orientation.
(XLSX)

S1 Data All numerical input data for main and supporting figures. (XLSX)

S1 Raw Images Original images for blots and gels. (PDF)

## Abbreviations

Azi: *p*-azidophenylalanine
CAT: chloramphenicol resistance cassette (chloramphenicol acyltransferase)
co-IP: co-immunoprecipitation
DHFR-TS: dihydrofolate reductase-thymidylate synthase
DMEM: Dulbecco’s Modified Eagle Medium
EloML: Elongated Machine Learning
FUDR: 5-fluorodeoxyuridine
GWCS: genome-wide CRISPR screen
HA: hemagglutinin
HDR: homology directed repair
HFF: human foreskin fibroblast
hpi: hours post infection
HXGPRT: hypoxanthine-xanthine-guanine phosphoribosyl transferase
IFA: immunofluorescence assay
ILP1: IMC localizing protein 1
IMC: inner membrane complex
ISP1: IMC sub-compartment protein 1
MIP: maximum intensity projection
PLV/VAC: plant-like vacuolar compartment
smMyc: spaghetti monster Myc
SPN: subpellicular network
TEM: transmission electron microscopy
UAA: unnatural amino acid

## References

[1] Gould SB, Tham W-H, Cowman AF, McFadden GI, Waller RF. Alveolins, a New Family of Cortical Proteins that Define the Protist Infrakingdom Alveolata. Mol Biol Evol. 2008;25:1219–1230. doi: 10.1093/molbev/msn070 18359944

[2] Levine ND, Corliss JO, Cox FEG, Deroux G, Grain J, Honigberg BM, et al. A Newly Revised Classification of the Protozoa*. J Protozool. 1980;27:37–58. doi: 10.1111/j.1550-7408.1980.tb04228.x 6989987

[3] Mackintosh CL, Beeson JG, Marsh K. Clinical features and pathogenesis of severe malaria. Trends Parasitol. 2004;20:597–603. doi: 10.1016/j.pt.2004.09.006 15522670

[4] Hill DE, Chirukandoth S, Dubey JP. Biology and epidemiology of Toxoplasma gondii in man and animals. Anim Health Res Rev. 2005;6:41–61. doi: 10.1079/ahr2005100 16164008

[5] Bouzid M, Hunter PR, Chalmers RM, Tyler KM. Cryptosporidium Pathogenicity and Virulence. Clin Microbiol Rev. 2013;26:115–134. doi: 10.1128/CMR.00076-12 23297262 PMC3553671

[6] Dubey JP. Review of *Neospora caninum* and neosporosis in animals. Korean J Parasitol. 2003;41:1–16. doi: 10.3347/kjp.2003.41.1.1 12666725 PMC2717477

[7] Daugschies A, Najdrowski M. Eimeriosis in Cattle: Current Understanding. J Vet Med B. 2005;52:417–427. doi: 10.1111/j.1439-0450.2005.00894.x 16364016

[8] Frénal K, Dubremetz J-F, Lebrun M, Soldati-Favre D. Gliding motility powers invasion and egress in Apicomplexa. Nat Rev Microbiol. 2017;15:645–660. doi: 10.1038/nrmicro.2017.86 28867819

[9] Harding CR, Meissner M. The inner membrane complex through development of *T oxoplasma gondii* and *P lasmodium*: The IMC in *Plasmodium* and *Toxoplasma*. Cell Microbiol. 2014;16:632–641. doi: 10.1111/cmi.12285 24612102 PMC4286798

[10] Francia ME, Striepen B. Cell division in apicomplexan parasites. Nat Rev Microbiol. 2014;12:125–136. doi: 10.1038/nrmicro3184 24384598

[11] Ferreira JL, Heincke D, Wichers JS, Liffner B, Wilson DW, Gilberger T-W. The Dynamic Roles of the Inner Membrane Complex in the Multiple Stages of the Malaria Parasite. Front Cell Infect Microbiol. 2021;10:611801. doi: 10.3389/fcimb.2020.611801 33489940 PMC7820811

[12] Back PS, O’Shaughnessy WJ, Moon AS, Dewangan PS, Hu X, Sha J, et al. Ancient MAPK ERK7 is regulated by an unusual inhibitory scaffold required for Toxoplasma apical complex biogenesis. PNAS. 2020;117:12164–12173. doi: 10.1073/pnas.1921245117 32409604 PMC7275706

[13] Tosetti N, Dos Santos Pacheco N, Bertiaux E, Maco B, Bournonville L, Hamel V, et al. Essential function of the alveolin network in the subpellicular microtubules and conoid assembly in Toxoplasma gondii. Silvie O, Akhmanova A, Tewari R, editors. Elife. 2020;9:e56635. doi: 10.7554/eLife.56635 32379047 PMC7228768

[14] O’Shaughnessy WJ, Hu X, Henriquez SA, Reese ML. Toxoplasma ERK7 protects the apical complex from premature degradation. J Cell Biol. 2023;222: e202209098. doi: 10.1083/jcb.202209098 37027006 PMC10083718

[15] Mann T, Beckers C. Characterization of the subpellicular network, a filamentous membrane skeletal component in the parasite Toxoplasma gondii. Mol Biochem Parasitol. 2001;115:257–268. doi: 10.1016/s0166-6851(01)00289-4 11420112

[16] Gubbels M-J, Wieffer M, Striepen B. Fluorescent protein tagging in Toxoplasma gondii: identification of a novel inner membrane complex component conserved among Apicomplexa. Mol Biochem Parasitol. 2004;137:99–110. doi: 10.1016/j.molbiopara.2004.05.007 15279956

[17] Hu K, Johnson J, Florens L, Fraunholz M, Suravajjala S, DiLullo C, et al. Cytoskeletal Components of an Invasion Machine—The Apical Complex of Toxoplasma gondii. PLoS Pathog. 2006;2:e13. doi: 10.1371/journal.ppat.0020013 16518471 PMC1383488

[18] Anderson-White BR, Ivey FD, Cheng K, Szatanek T, Lorestani A, Beckers CJ, et al. A family of intermediate filament-like proteins is sequentially assembled into the cytoskeleton of Toxoplasma gondii. Cell Microbiol. 2011;13:18–31. doi: 10.1111/j.1462-5822.2010.01514.x 20698859 PMC3005026

[19] Lorestani A, Ivey FD, Thirugnanam S, Busby MA, Marth GT, Cheeseman IM, et al. Targeted proteomic dissection of *Toxoplasma* cytoskeleton sub-compartments using MORN1. Cytoskeleton. 2012;69:1069–1085. doi: 10.1002/cm.21077 23027733 PMC3566231

[20] Chen AL, Kim EW, Toh JY, Vashisht AA, Rashoff AQ, Van C, et al. Novel Components of the Toxoplasma Inner Membrane Complex Revealed by BioID. MBio. 2015;6:e02357–14. doi: 10.1128/mBio.02357-14 25691595 PMC4337574

[21] Chen AL, Moon AS, Bell HN, Huang AS, Vashisht AA, Toh JY, et al. Novel insights into the composition and function of the Toxoplasma IMC sutures. Cell Microbiol. 2017;19:e12678. doi: 10.1111/cmi.12678 27696623 PMC5909696

[22] Choi CP, Moon AS, Back PS, Jami-Alahmadi Y, Vashisht AA, Wohlschlegel JA, et al. A photoactivatable crosslinking system reveals protein interactions in the Toxoplasma gondii inner membrane complex. PLoS Biol. 2019;17:e3000475. doi: 10.1371/journal.pbio.3000475 31584943 PMC6795473

[23] Chin JW, Cropp TA, Anderson JC, Mukherji M, Zhang Z, Schultz PG. An Expanded Eukaryotic Genetic Code. Science. 2003;301:964–967. doi: 10.1126/science.1084772 12920298

[24] Dubey R, Harrison B, Dangoudoubiyam S, Bandini G, Cheng K, Kosber A, et al. Differential Roles for Inner Membrane Complex Proteins across Toxoplasma gondii and Sarcocystis neurona Development. mSphere. 2017;2:e00409–17. doi: 10.1128/mSphere.00409-17 29062899 PMC5646244

[25] Coghlan MP, Tremp AZ, Saeed S, Vaughan CK, Dessens JT. Distinct Functional Contributions by the Conserved Domains of the Malaria Parasite Alveolin IMC1h. Frontiers in Cellular and Infection Microbiology. 2019;9. Available from: https://www.frontiersin.org/articles/10.3389/fcimb.2019.00266 31428588 10.3389/fcimb.2019.00266 PMC6689960

[26] Sidik SM, Huet D, Ganesan SM, Huynh M-H, Wang T, Nasamu AS, et al. A Genome-wide CRISPR Screen in Toxoplasma Identifies Essential Apicomplexan Genes. Cell. 2016;166:1423–1435.e12. doi: 10.1016/j.cell.2016.08.019 27594426 PMC5017925

[27] Harb OS, Roos DS. ToxoDB: Functional Genomics Resource for Toxoplasma and Related Organisms. Methods Mol Biol. 2020;2071:27–47. doi: 10.1007/978-1-4939-9857-9_2 31758445

[28] Chern JH, Pasquarelli RR, Moon AS, Chen AL, Sha J, Wohlschlegel JA, et al. A Novel Toxoplasma Inner Membrane Complex Suture-Associated Protein Regulates Suture Protein Targeting and Colocalizes with Membrane Trafficking Machinery. MBio. 2021;12:e02455–21. doi: 10.1128/mBio.02455-21 34634933 PMC8510555

[29] Back PS, Moon AS, Pasquarelli RR, Bell HN, Torres JA, Chen AL, et al. IMC29 Plays an Important Role in Toxoplasma Endodyogeny and Reveals New Components of the Daughter-Enriched IMC Proteome. MBio. 2023;14:e03042–22. doi: 10.1128/mbio.03042-22 36622147 PMC9973257

[30] Leung JM, Rould MA, Konradt C, Hunter CA, Ward GE. Disruption of TgPHIL1 Alters Specific Parameters of Toxoplasma gondii Motility Measured in a Quantitative, Three-Dimensional Live Motility Assay. PLoS ONE. 2014;9:e85763. doi: 10.1371/journal.pone.0085763 24489670 PMC3906025

[31] Engelberg K, Bechtel T, Michaud C, Weerapana E, Gubbels M-J. Proteomic characterization of the Toxoplasma gondii cytokinesis machinery portrays an expanded hierarchy of its assembly and function. Nat Commun. 2022;13:4644. doi: 10.1038/s41467-022-32151-0 35941170 PMC9360017

[32] Roumégous C, Abou Hammoud A, Fuster D, Dupuy J-W, Blancard C, Salin B, et al. Identification of new components of the basal pole of Toxoplasma gondii provides novel insights into its molecular organization and functions. Front Cell Infect Microbiol. 2022;12:1010038. doi: 10.3389/fcimb.2022.1010038 36310866 PMC9613666

[33] Drozdetskiy A, Cole C, Procter J, Barton GJ. JPred4: a protein secondary structure prediction server. Nucleic Acids Res. 2015;43:W389–W394. doi: 10.1093/nar/gkv332 25883141 PMC4489285

[34] Nath Jha A, Vishveshwara S, Banavar JR. Amino acid interaction preferences in proteins. Protein Sci. 2010;19:603–616. doi: 10.1002/pro.339 20073083 PMC2866284

[35] Oliveira Souza RO, Jacobs KN, Back PS, Bradley PJ, Arrizabalaga G. IMC10 and LMF1 mediate mitochondrial morphology through mitochondrion–pellicle contact sites in Toxoplasma gondii. J Cell Sci. 2022;135:jcs260083. doi: 10.1242/jcs.260083 36314270 PMC9845740

[36] Barkhuff WD, Gilk SD, Whitmarsh R, Tilley LD, Hunter C, Ward GE. Targeted Disruption of TgPhIL1 in Toxoplasma gondii Results in Altered Parasite Morphology and Fitness. PLoS ONE. 2011;6:e23977. doi: 10.1371/journal.pone.0023977 21901148 PMC3162014

[37] Tilley LD, Krishnamurthy S, Westwood NJ, Ward GE. Identification of TgCBAP, a Novel Cytoskeletal Protein that Localizes to Three Distinct Subcompartments of the Toxoplasma gondii Pellicle. PLoS ONE. 2014;9:e98492. doi: 10.1371/journal.pone.0098492 24887026 PMC4041824

[38] Lentini G, Kong-Hap M, El Hajj H, Francia M, Claudet C, Striepen B, et al. Identification and characterization of Toxoplasma SIP, a conserved apicomplexan cytoskeleton protein involved in maintaining the shape, motility and virulence of the parasite. Cell Microbiol. 2015;17:62–78. doi: 10.1111/cmi.12337 25088010 PMC4639917

[39] Harding CR, Gow M, Kang JH, Shortt E, Manalis SR, Meissner M, et al. Alveolar proteins stabilize cortical microtubules in Toxoplasma gondii. Nat Commun. 2019;10:401. doi: 10.1038/s41467-019-08318-7 30674885 PMC6344517

[40] Khater EI, Sinden RE, Dessens JT. A malaria membrane skeletal protein is essential for normal morphogenesis, motility, and infectivity of sporozoites. J Cell Biol. 2004;167:425–432. doi: 10.1083/jcb.200406068 15533999 PMC2172497

[41] Tremp AZ, Khater EI, Dessens JT. IMC1b Is a Putative Membrane Skeleton Protein Involved in Cell Shape, Mechanical Strength, Motility, and Infectivity of Malaria Ookinetes *. J Biol Chem. 2008;283:27604–27611. doi: 10.1074/jbc.M801302200 18650444 PMC2562075

[42] Tremp AZ, Dessens JT. Malaria IMC1 Membrane Skeleton Proteins Operate Autonomously and Participate in Motility Independently of Cell Shape *. J Biol Chem. 2011;286:5383–5391. doi: 10.1074/jbc.M110.187195 21098480 PMC3037651

[43] Cepeda Diaz AK, Rudlaff RM, Farringer M, Dvorin JD. Essential function of alveolin PfIMC1g in the Plasmodium falciparum asexual blood stage. MBio. 2023;14:e01507–23. doi: 10.1128/mbio.01507-23 37712738 PMC10653860

[44] Tremp AZ, Carter V, Saeed S, Dessens JT. Morphogenesis of Plasmodium zoites is uncoupled from tensile strength. Mol Microbiol. 2013;89:552–564. doi: 10.1111/mmi.12297 23773015 PMC3912903

[45] El-Haddad H, Przyborski JM, Kraft LGK, McFadden GI, Waller RF, Gould SB. Characterization of TtALV2, an Essential Charged Repeat Motif Protein of the Tetrahymena thermophila Membrane Skeleton. Eukaryot Cell. 2013;12:932–940. doi: 10.1128/EC.00050-13 23606287 PMC3675985

[46] Ren J, Wen L, Gao X, Jin C, Xue Y, Yao X. CSS-Palm 2.0: an updated software for palmitoylation sites prediction. Protein Eng Des Sel. 2008;21:639–644. doi: 10.1093/protein/gzn039 18753194 PMC2569006

[47] Mann T, Gaskins E, Beckers C. Proteolytic Processing of TgIMC1 during Maturation of the Membrane Skeleton of Toxoplasma gondii. J Biol Chem. 2002;277:41240–41246. doi: 10.1074/jbc.M205056200 12177058

[48] Donald RGK, Roos DS. Stable molecular transformation of Toxoplasma gondii: a selectable dihydrofolate reductase-thymidylate synthase marker based on drug-resistance mutations in malaria. Proc Natl Acad Sci U S A. 1993;90:11703–11707. doi: 10.1073/pnas.90.24.11703 8265612 PMC48052

[49] Kim K, Soldati D, Boothroyd JC. Gene Replacement in Toxoplasma gondii with Chloramphenicol Acetyltransferase as Selectable Marker. Science. 1993;262:911–914. doi: 10.1126/science.8235614 8235614

[50] Donald RGK, Carter D, Ullman B, Roos DS. Insertional Tagging, Cloning, and Expression of the Toxoplasma gondii Hypoxanthine-Xanthine-Guanine Phosphoribosyltransferase Gene: USE AS A SELECTABLE MARKER FOR STABLE TRANSFORMATION*. J Biol Chem. 1996;271:14010–14019. doi: 10.1074/jbc.271.24.14010 8662859

[51] Donald RG, Roos DS. Insertional mutagenesis and marker rescue in a protozoan parasite: cloning of the uracil phosphoribosyltransferase locus from Toxoplasma gondii. Proc Natl Acad Sci U S A. 1995;92:5749–5753. doi: 10.1073/pnas.92.12.5749 7777580 PMC41774

[52] Sidik SM, Hackett CG, Tran F, Westwood NJ, Lourido S. Efficient Genome Engineering of Toxoplasma gondii Using CRISPR/Cas9. PLoS ONE. 2014;9:e100450. doi: 10.1371/journal.pone.0100450 24971596 PMC4074098

[53] Bastin P, Bagherzadeh A, Matthews KR, Gull K. A novel epitope tag system to study protein targeting and organelle biogenesis in Trypanosoma brucei. Mol Biochem Parasitol. 1996;77:235–239. doi: 10.1016/0166-6851(96)02598-4 8813669

[54] Wichroski MJ, Melton JA, Donahue CG, Tweten RK, Ward GE. Clostridium septicum Alpha-Toxin Is Active against the Parasitic Protozoan Toxoplasma gondii and Targets Members of the SAG Family of Glycosylphosphatidylinositol-Anchored Surface Proteins. Infect Immun. 2002;70:4353–4361. doi: 10.1128/IAI.70.8.4353-4361.2002 12117945 PMC128134

[55] Back PS, O’Shaughnessy WJ, Moon AS, Dewangan PS, Reese ML, Bradley PJ. Multivalent Interactions Drive the Toxoplasma AC9:AC10:ERK7 Complex To Concentrate ERK7 in the Apical Cap. MBio. 2022;13:e02864–21. doi: 10.1128/mbio.02864-21 35130732 PMC8822341

[56] Beck JR, Rodriguez-Fernandez IA, de Leon JC, Huynh M-H, Carruthers VB, Morrissette NS, et al. A novel family of Toxoplasma IMC proteins displays a hierarchical organization and functions in coordinating parasite division. PLoS Pathog. 2010;6:e1001094. doi: 10.1371/journal.ppat.1001094 20844581 PMC2936552

[57] Huynh M-H, Carruthers VB. A Toxoplasma gondii Ortholog of Plasmodium GAMA Contributes to Parasite Attachment and Cell Invasion. mSphere. 2016 [cited 2022 Jan 18]. doi: 10.1128/mSphere.00012-16 27303694 PMC4863602

[58] Rome ME, Beck JR, Turetzky JM, Webster P, Bradley PJ. Intervacuolar Transport and Unique Topology of GRA14, a Novel Dense Granule Protein in Toxoplasma gondii. Infect Immun. 2008 [cited 2022 Jan 18]. doi: 10.1128/IAI.00782-08 18765740 PMC2573327

[59] Bradley PJ, Ward C, Cheng SJ, Alexander DL, Coller S, Coombs GH, et al. Proteomic Analysis of Rhoptry Organelles Reveals Many Novel Constituents for Host-Parasite Interactions in Toxoplasma gondii*. J Biol Chem. 2005;280:34245–34258. doi: 10.1074/jbc.M504158200 16002398

[60] Nadipuram SM, Kim EW, Vashisht AA, Lin AH, Bell HN, Coppens I, et al. In Vivo Biotinylation of the Toxoplasma Parasitophorous Vacuole Reveals Novel Dense Granule Proteins Important for Parasite Growth and Pathogenesis. MBio. 2016;7:e00808–16. doi: 10.1128/mBio.00808-16 27486190 PMC4981711

[61] Frénal K, Marq J-B, Jacot D, Polonais V, Soldati-Favre D. Plasticity between MyoC- and MyoA-Glideosomes: An Example of Functional Compensation in Toxoplasma gondii Invasion. PLoS Pathog. 2014;10:e1004504. doi: 10.1371/journal.ppat.1004504 25393004 PMC4231161

[62] DeRocher AE, Coppens I, Karnataki A, Gilbert LA, Rome ME, Feagin JE, et al. A Thioredoxin Family Protein of the Apicoplast Periphery Identifies Abundant Candidate Transport Vesicles in Toxoplasma gondii. Eukaryot Cell. 2008 [cited 2022 Jan 18]. doi: 10.1128/EC.00081-08 18586952 PMC2547066

[63] Francia ME, Wicher S, Pace DA, Sullivan J, Moreno SNJ, Arrizabalaga G. A Toxoplasma gondii protein with homology to intracellular type Na+/H+ exchangers is important for osmoregulation and invasion. Exp Cell Res. 2011;317:1382–1396. doi: 10.1016/j.yexcr.2011.03.020 21501607 PMC3096714

[64] Dunn JD, Ravindran S, Kim S-K, Boothroyd JC. The Toxoplasma gondii Dense Granule Protein GRA7 Is Phosphorylated upon Invasion and Forms an Unexpected Association with the Rhoptry Proteins ROP2 and ROP4. Infect Immun. 2008;76:5853–5861. doi: 10.1128/IAI.01667-07 18809661 PMC2583583

[65] Kafsack BFC, Pena JDO, Coppens I, Ravindran S, Boothroyd JC, Carruthers VB. Rapid Membrane Disruption by a Perforin-Like Protein Facilitates Parasite Exit from Host Cells. Science. 2009;323:530–533. doi: 10.1126/science.1165740 19095897 PMC2662845

[66] Carruthers VB, Sibley LD. Mobilization of intracellular calcium stimulates microneme discharge in *Toxoplasma gondii*. Mol Microbiol. 1999;31:421–428. doi: 10.1046/j.1365-2958.1999.01174.x 10027960

