# Supplementary figures and images for: Alveolin proteins in the Toxoplasma inner membrane complex form a highly interconnected structure that maintains parasite shape and replication
Source: PLoS Biol. 2024 Sep 12;22(9):e3002809. doi: 10.1371/journal.pbio.3002809 (PMC11421793; doi:10.1371/journal.pbio.3002809)

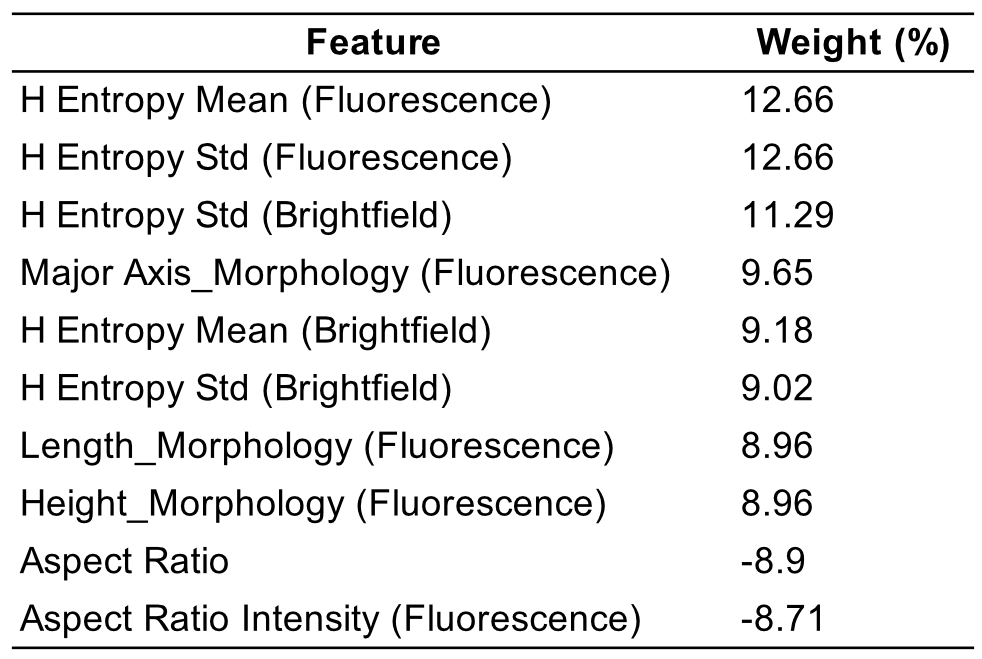

Supplement: S1 Fig — Table of features used in the Elongated ML classifier and their weight. (TIFF) [file pbio.3002809.s001.tiff]

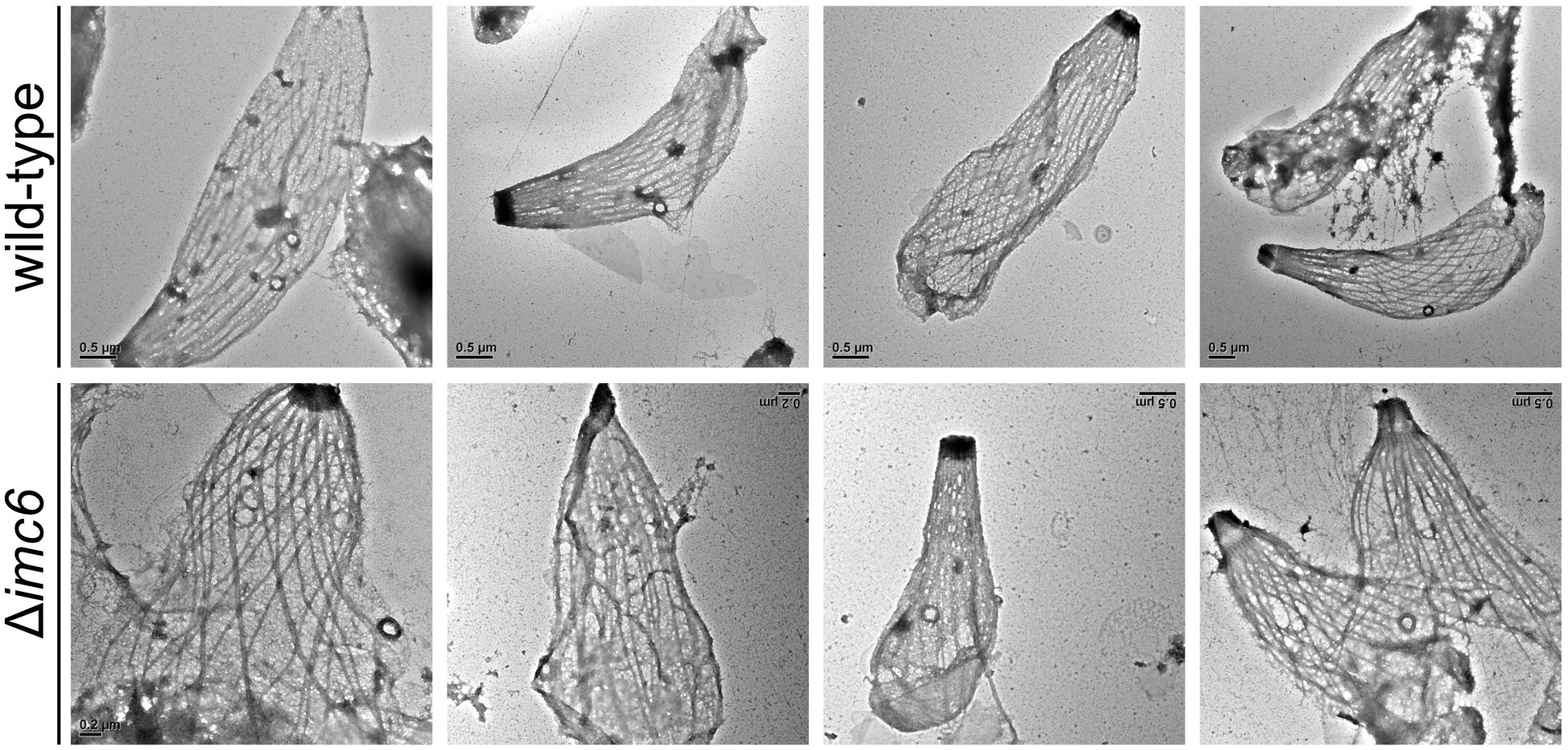

Supplement: S2 Fig — Detergent extracted and negatively stained parasites, highlighting the microtubules and subpellicular network. (TIFF) [file pbio.3002809.s002.tiff]

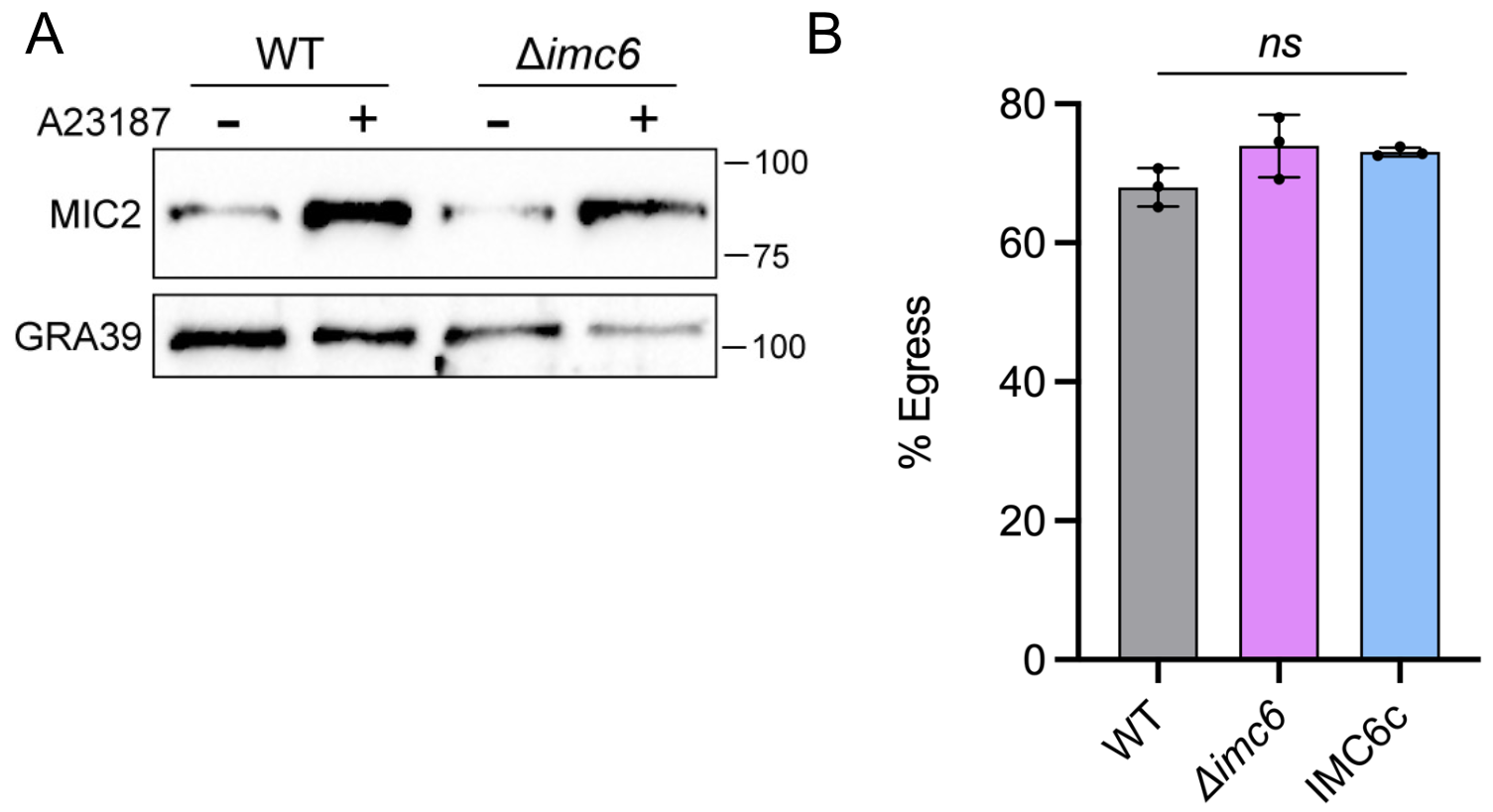

Supplement: S3 Fig — (A) Western blot of secreted proteins from WT and Δimc6 parasites. The calcium ionophore A23187 was used to induce microneme secretion. MIC2 was detected with anti-MIC2, and the constitutively secreted dense granule protein GRA39 was used as a control, detected with anti-GRA39. (B) Calcium ionophore-induced egress assay, quantified by counting the number of vacuoles that have egressed or not. Data are plotted as the mean ± SD, and significance was calculated using two-tailed t tests. The raw data underlying this figure can be found in S1 Data. (TIFF) [file pbio.3002809.s003.tiff]

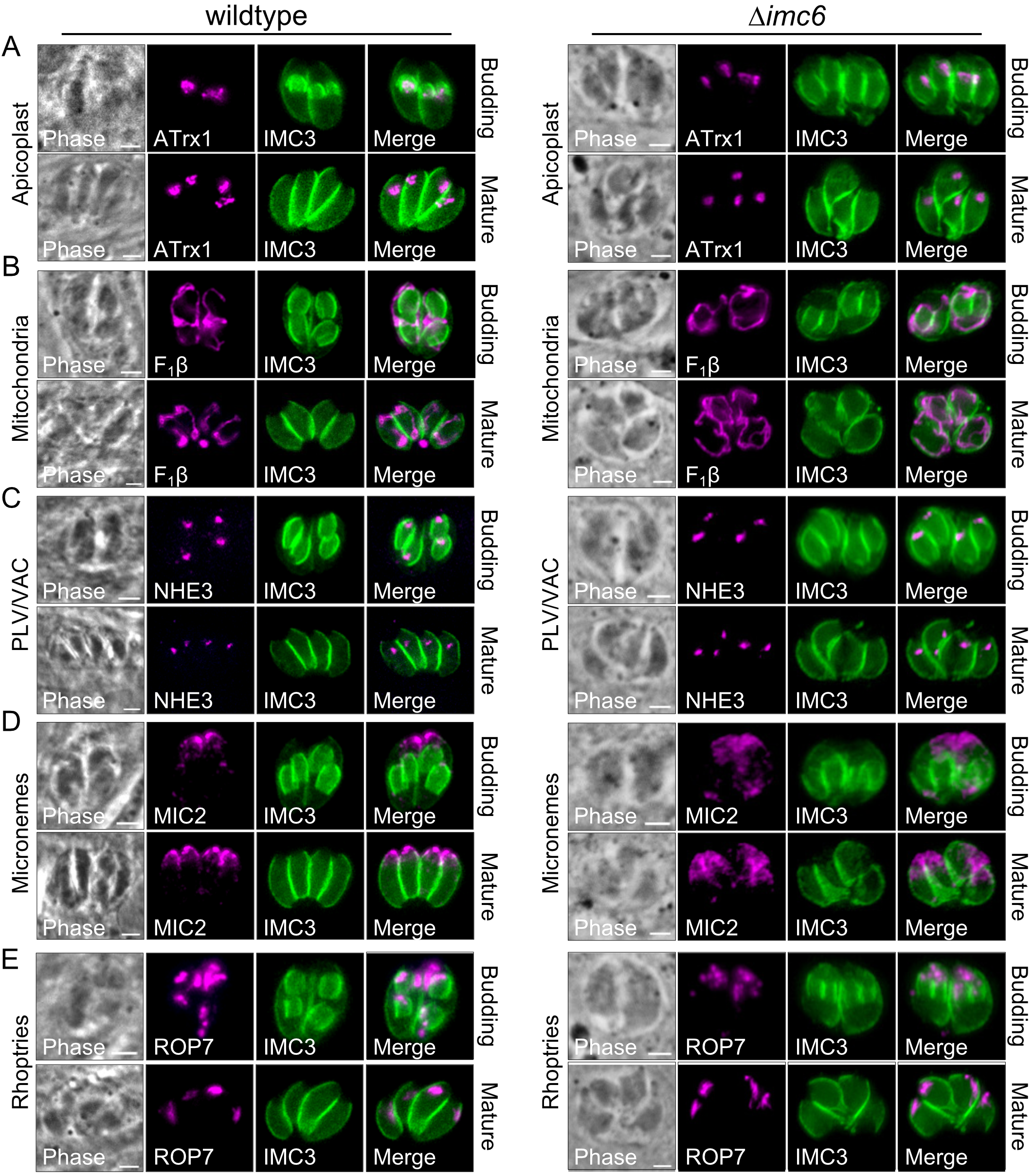

Supplement: S4 Fig — (A-E) IFAs of mature and budding wild-type or Δimc6 parasites, showing typical morphology of the indicated organelles. (A) The apicoplast was detected with anti-ATrx1 (magenta). (B) Mitochondria were detected with anti-F1β (magenta). (C) The PLV/VAC was detected with anti-NHE3 (magenta). (D) Micronemes were detected with anti-MIC2 (magenta). (D) Rhoptries were detected with anti-ROP7 (magenta). All IFAs were costained with anti-IMC3 (green). All scale bars are 2 μm. (TIFF) [file pbio.3002809.s004.tiff]

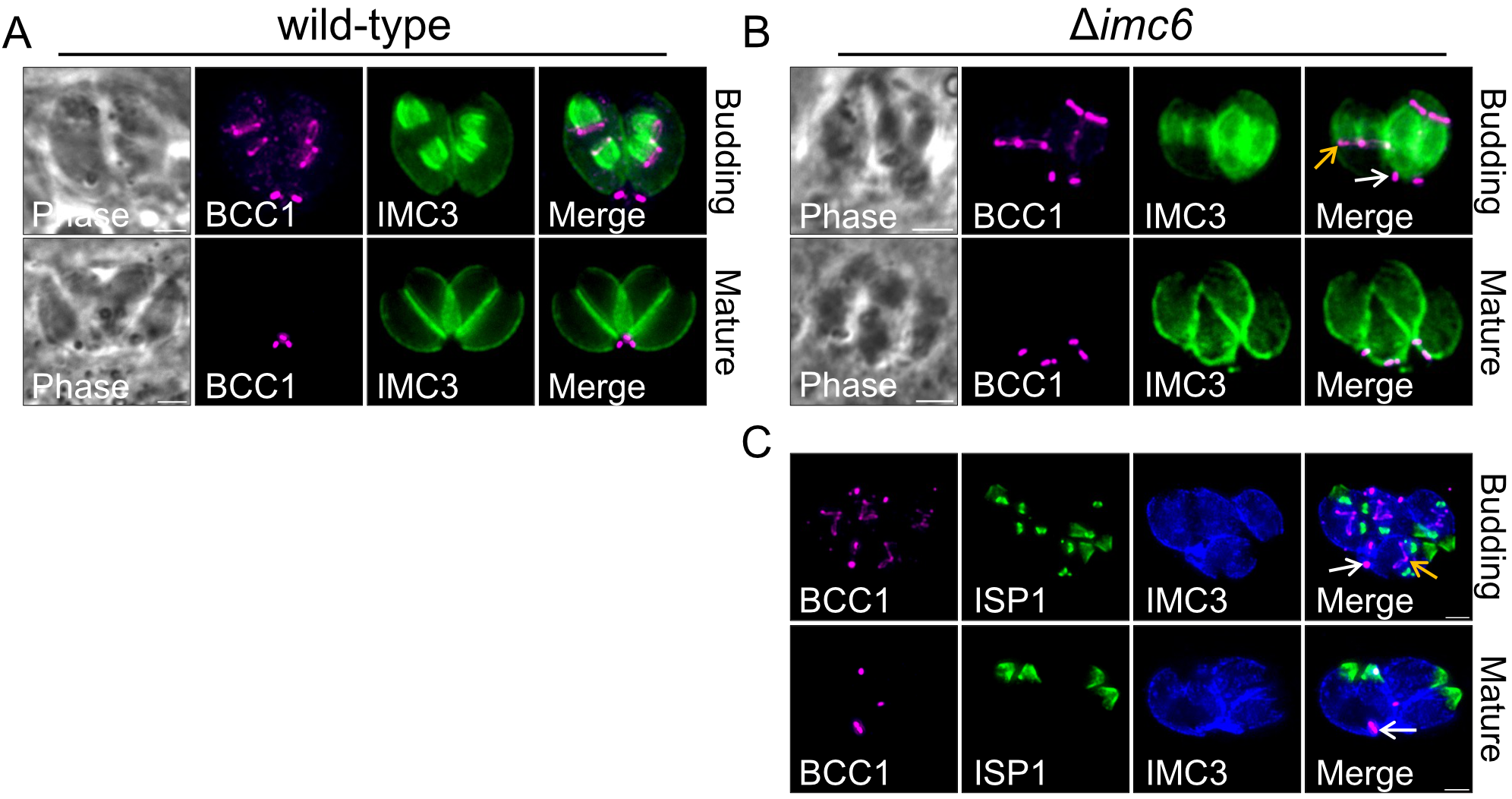

Supplement: S5 Fig — (A) IFAs of wild-type parasites show proper BCC1 localization. (B) IFAs of knockout parasites show proper BCC1 localization despite morphological and replication defects. White arrows point to maternal basal complexes and orange arrows point to daughter basal complexes. (C) IFAs of incompletely separated knockout parasites, which still show proper BCC1 localization. White arrows point to maternal basal complexes and orange arrows point to daughter basal complexes. BCC1 was detected with mouse anti-HA. All scale bars are 2 μm. (TIFF) [file pbio.3002809.s005.tiff]

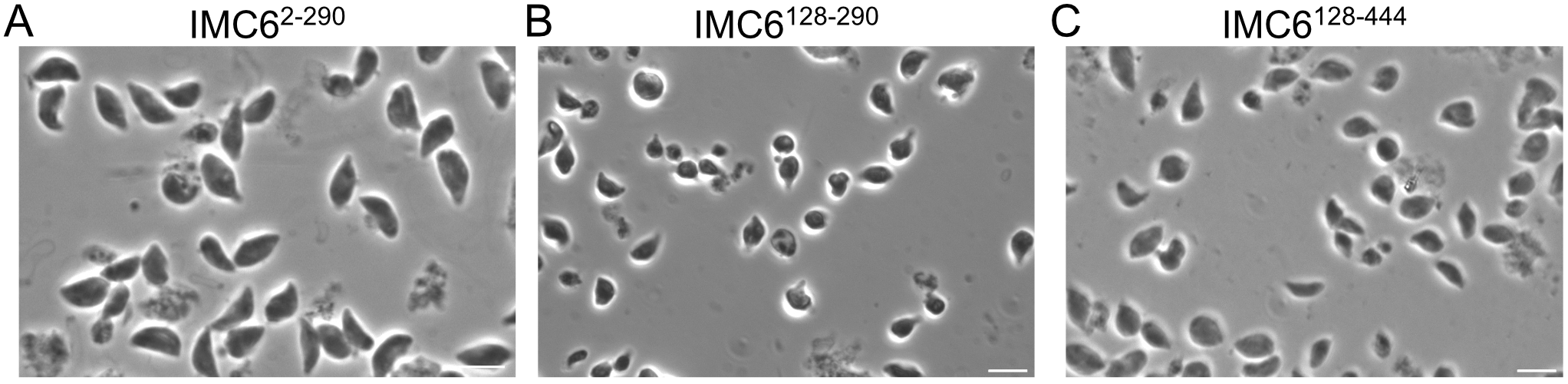

Supplement: S6 Fig — Phase contrast images of extracellular parasites show partial rescue of the shape defect. All scale bars are 5 μm. (TIFF) [file pbio.3002809.s006.tiff]
