# Supplementary material for: Alveolin proteins in the Toxoplasma inner membrane complex form a highly interconnected structure that maintains parasite shape and replication
Source: PLoS Biol. 2024 Sep 12;22(9):e3002809. doi: 10.1371/journal.pbio.3002809 (PMC11421793; doi:10.1371/journal.pbio.3002809)

S1 Raw images.

Raw images of western blots and gels for all figures and supporting figures.

Fig 1C. DNA agarose gel for IMC6 knockout verification.
Box denotes regions used in figure.

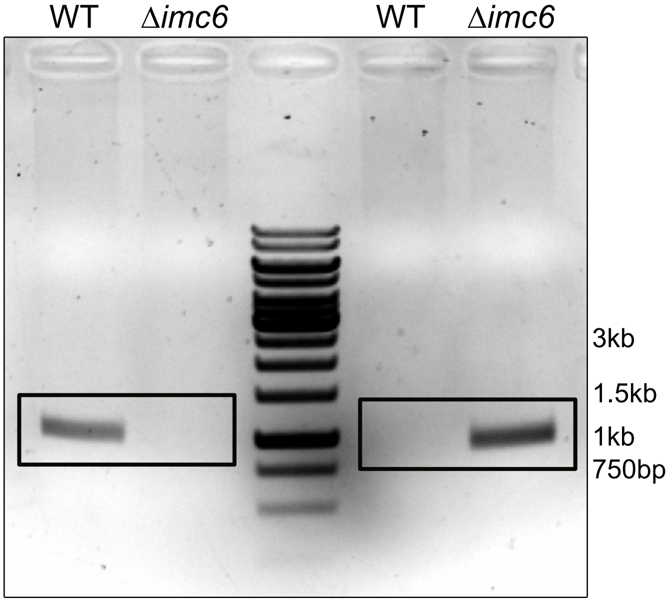

Fig 1F. Western blot to confirm absence and restoration of IMC6 expression in knockout and complemented strains.
Box denotes regions used in figure. Unboxed regions are unrelated to this figure.

A) Chemiluminescent signal used for anti-IMC6-HRP.
B) Chemiluminescent signal used for anti-ROP13-HRP.
C) Merged with colorimetric image showing molecular weight markers in kDa.

A

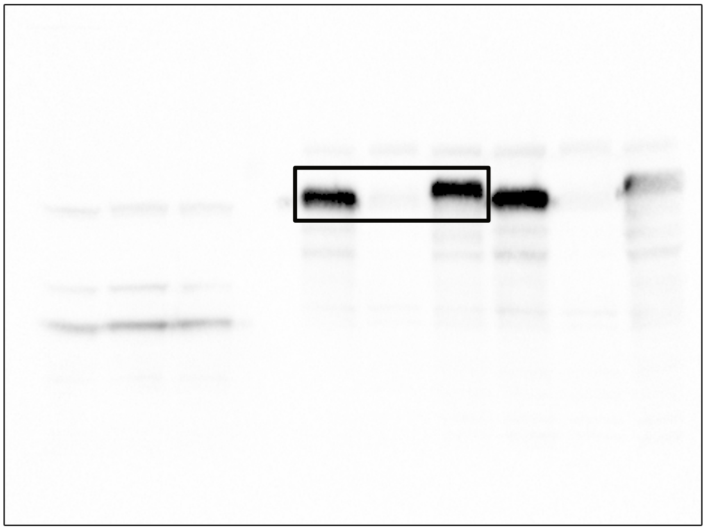

B

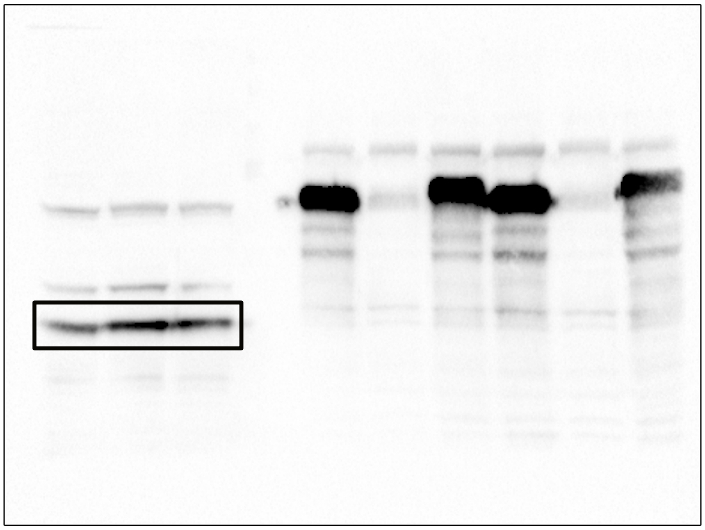

C

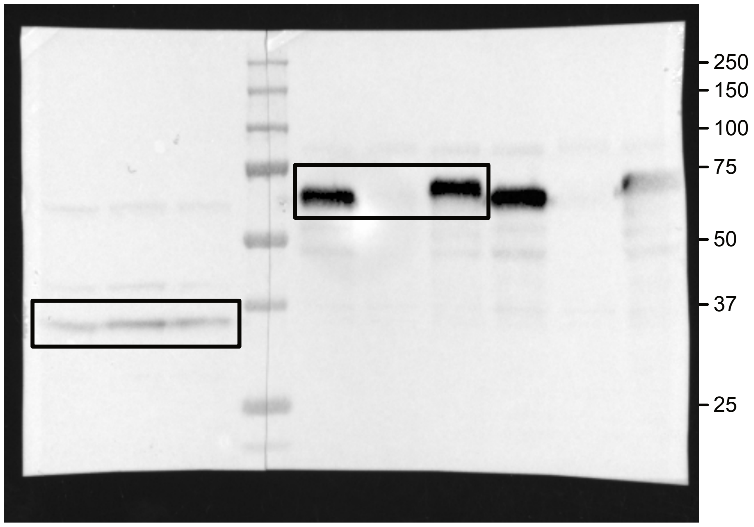

Fig 2C-D and Fig 5F-G. Images of gating used for imagestream flow cytometry analysis.

Images show gated populations, progressing from left to right until only single cells remained.

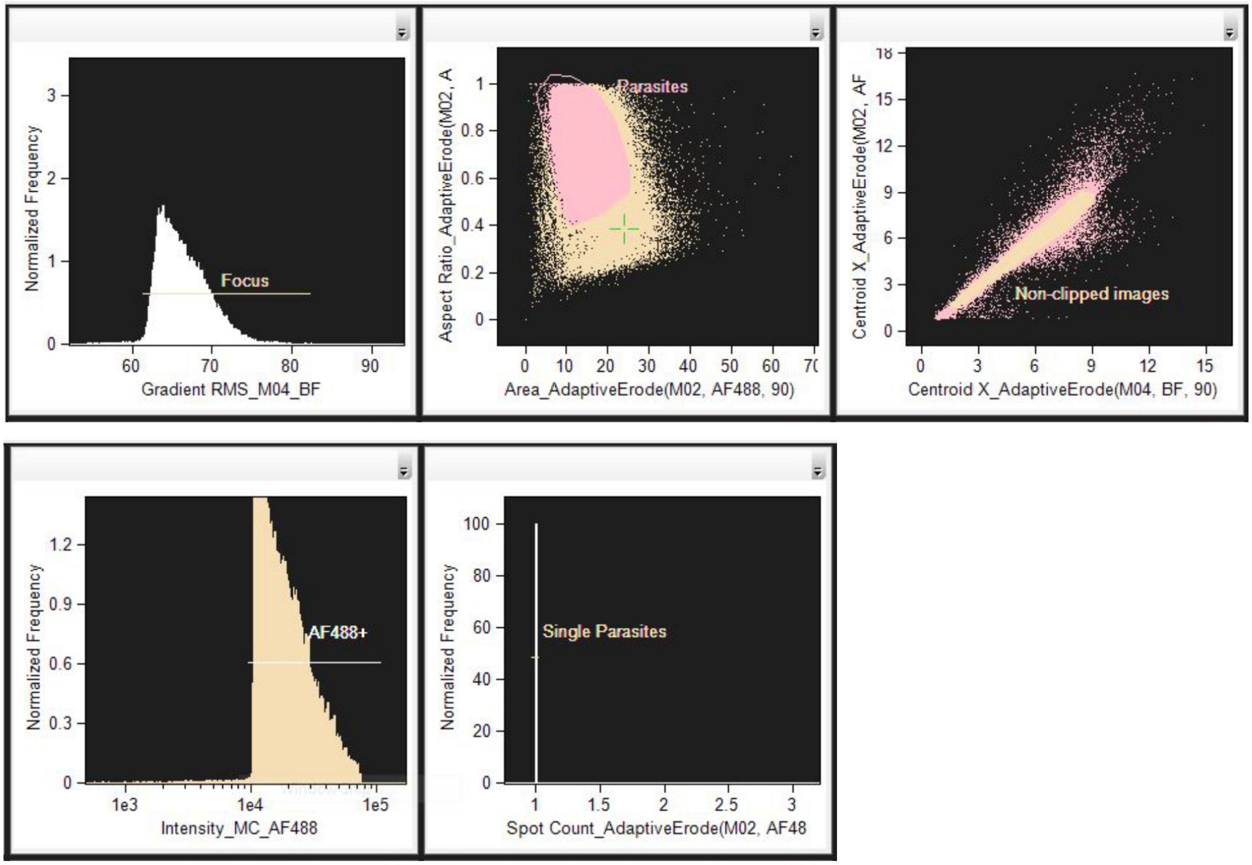

Fig 7. Western blots of crosslinked amber mutants across the alveolin domain.
All molecular weight markers are in kDa.

A) Fig 7B - chemiluminescent signal used for anti-HA-HRP.
B) Fig 7B - merge with colorimetric image showing molecular weight markers.
C) Fig 7C - chemiluminescent signal used for anti-HA-HRP.
D) Fig 7C - merge with colorimetric image showing molecular weight markers.
E) Fig 7D - chemiluminescent signal used for anti-HA-HRP.
F) Fig 7D - merge with colorimetric image showing molecular weight markers.

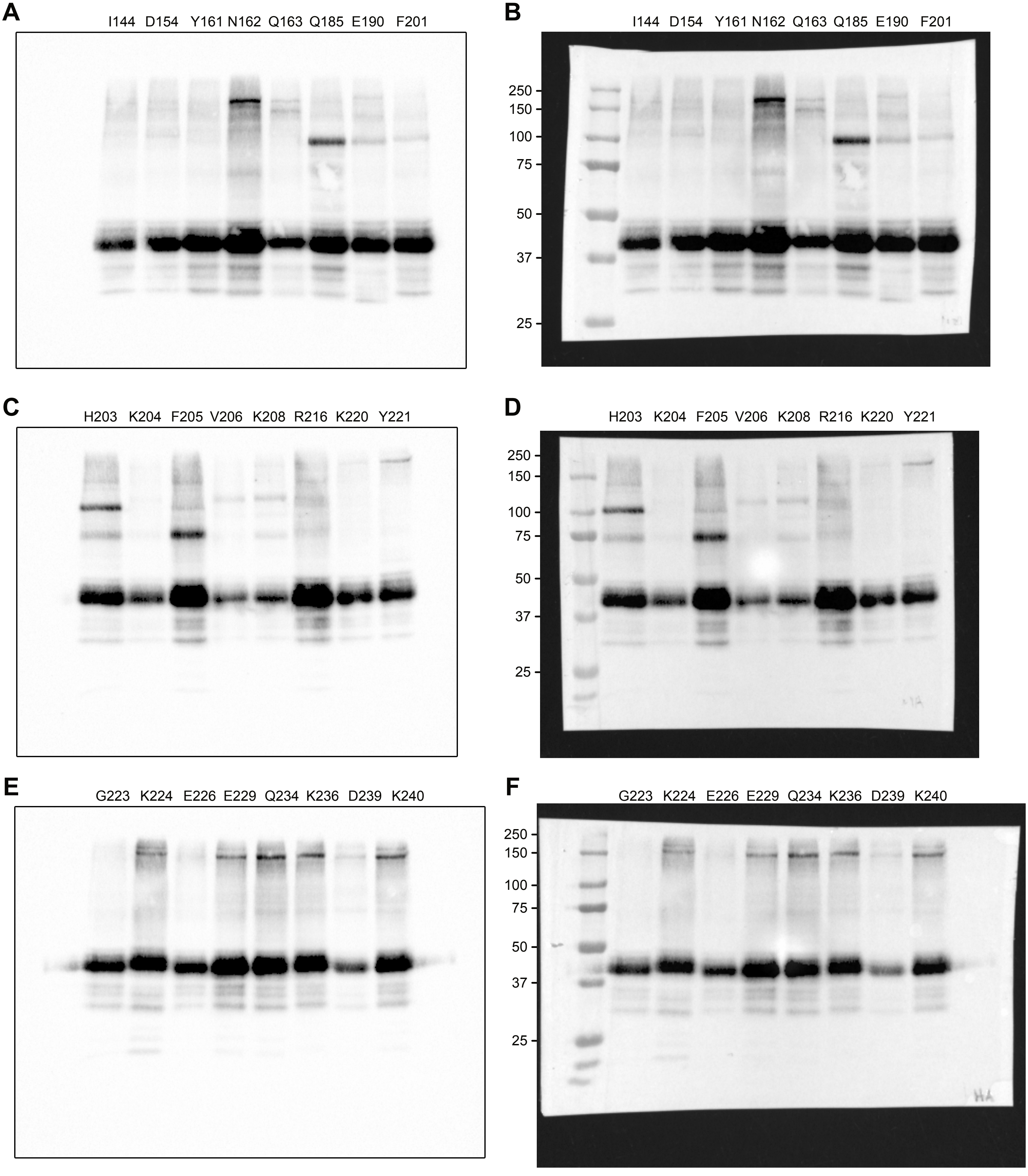

Fig 7. Western blots of crosslinked amber mutants across the alveolin domain.
All molecular weight markers are in kDa.

G) Fig 7E - chemiluminescent signal used for anti-HA-HRP.
H) Fig 7E - merge with colorimetric image showing molecular weight markers.
I) Fig 7F - chemiluminescent signal used for anti-HA-HRP.
J) Fig 7F - merge with colorimetric image showing molecular weight markers.

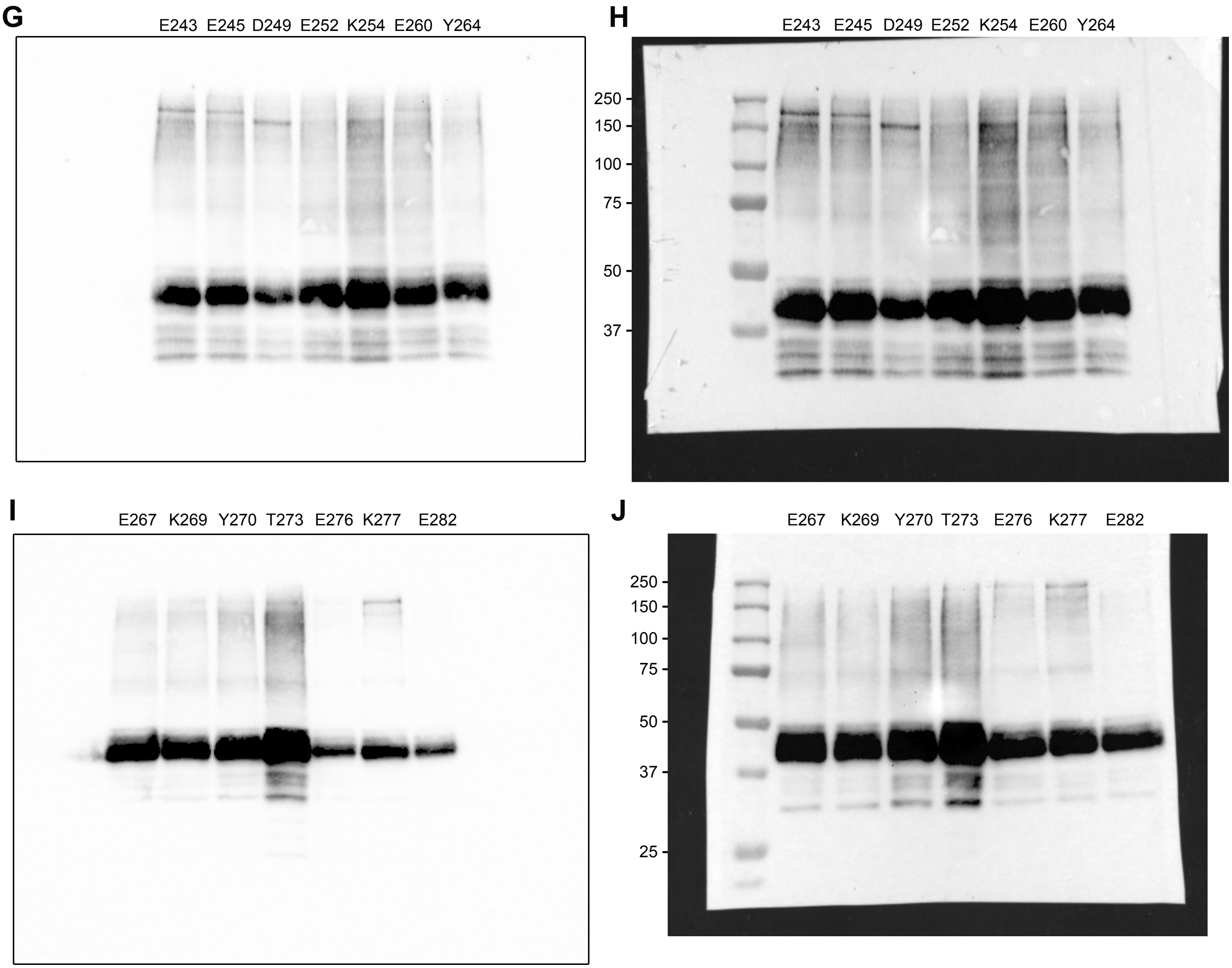

Fig 8. Western blots of IMC3-tagged and untagged amber mutants after crosslinking.
Box denotes regions used in figure. X denotes lanes unrelated to this figure.

A) Fig 8A - chemiluminescent signal of co-IP used to show anti-HA-HRP image.
B) Fig 8A - chemiluminescent signal of co-IP used to show anti-IMC3-HRP image.
C) Fig 8A - merged image showing molecular weight markers in kDa.

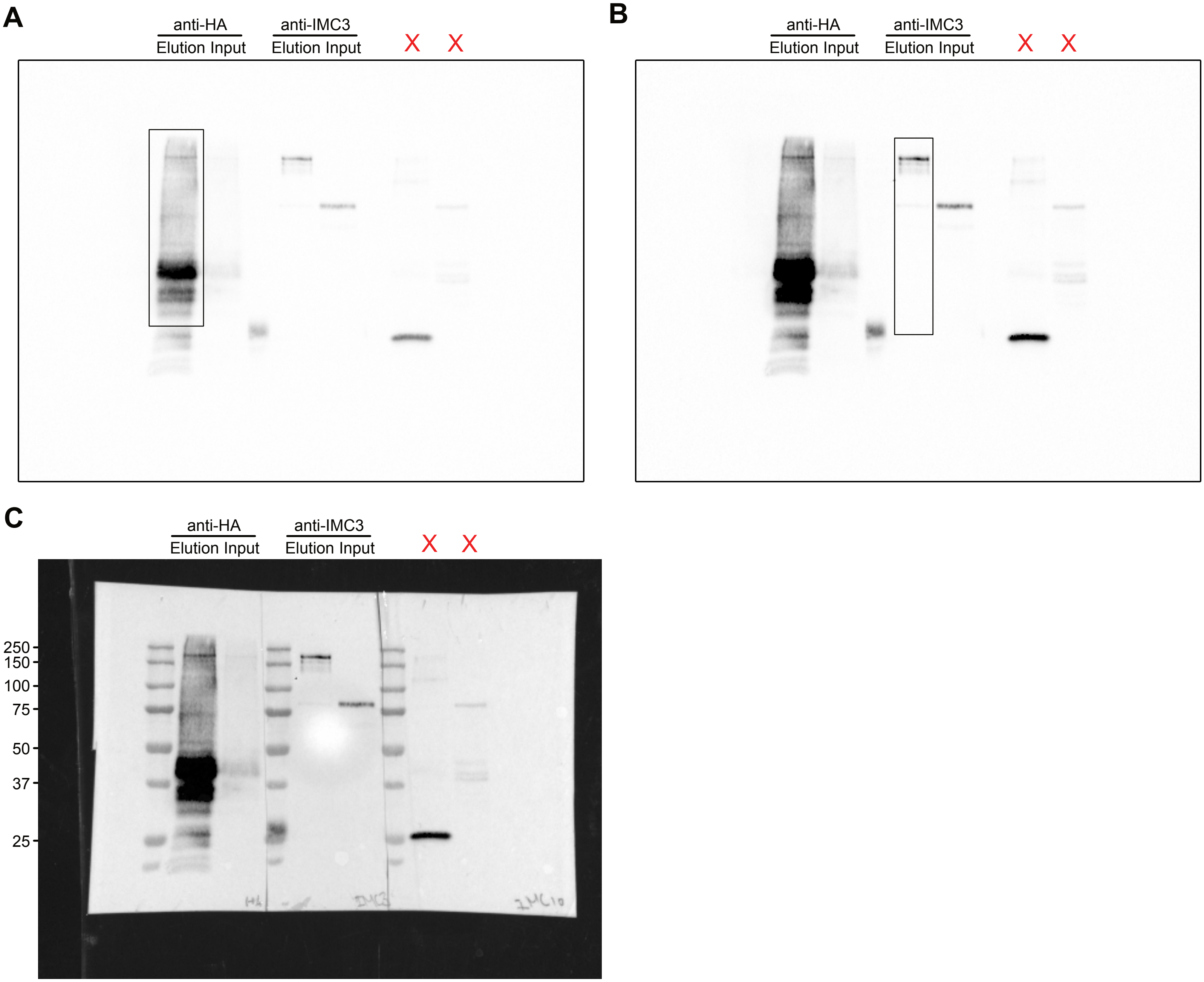

Fig 8. Western blots of IMC3-tagged and untagged amber mutants after crosslinking.
Box denotes regions used in figure. X denotes lanes unrelated to this figure.
A) Fig 8B - chemiluminescent signal of co-IP used to show anti-HA-HRP image.
B) Fig 8B - merged image with molecular weight markers.
C) Fig 8C - chemiluminescent signal of co-IP used to show anti-HA-HRP image.
D) Fig 8C - merged image with molecular weight markers.
E) Fig 8D - chemiluminescent signal of co-IP used to show anti-HA-HRP image.
F) Fig 8D - merged image with molecular weight markers.

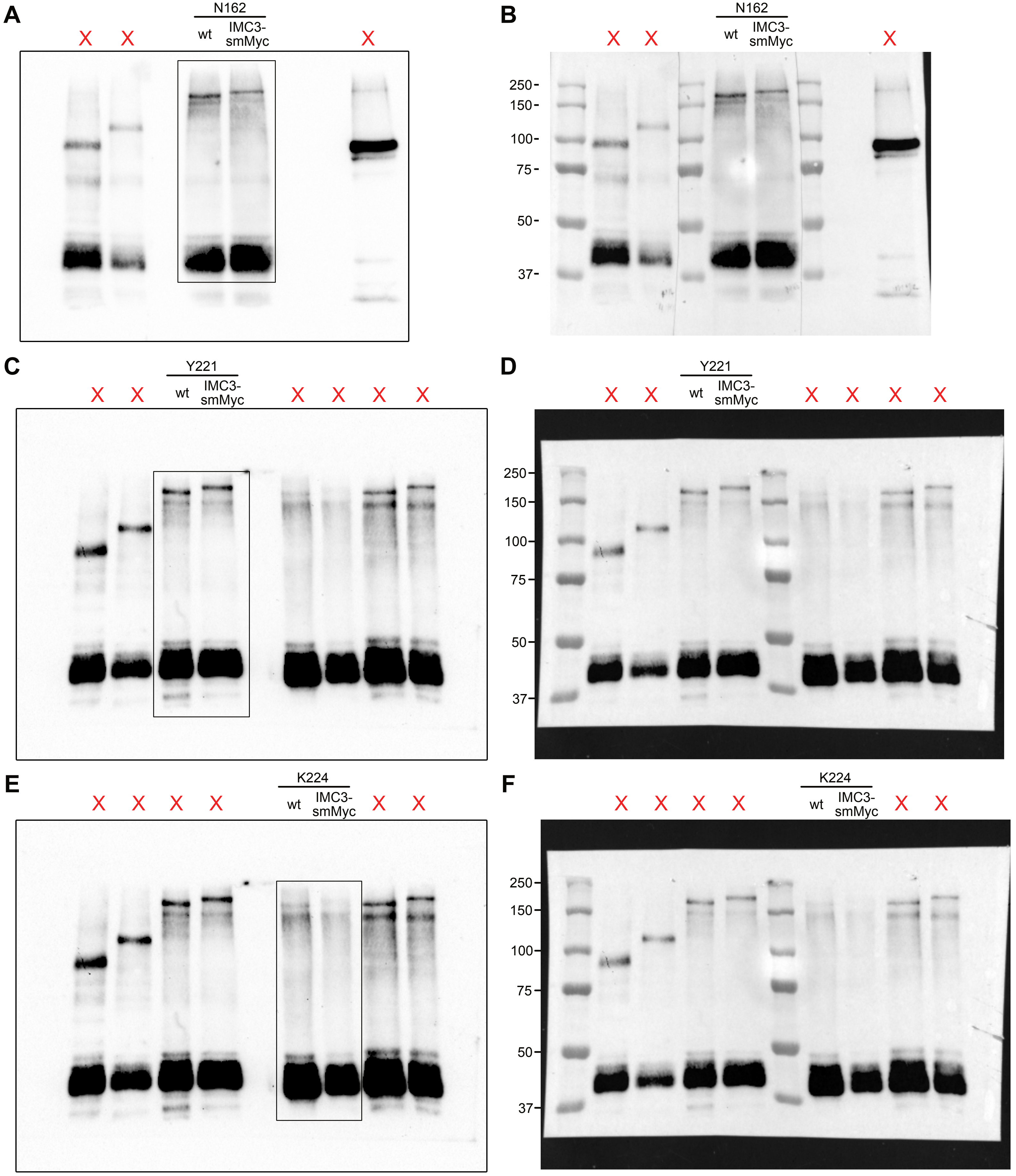

Fig 8. Western blots of IMC3-tagged and untagged amber mutants after crosslinking.
Box denotes regions used in figure. X denotes lanes unrelated to this figure.
A) Fig 8E - chemiluminescent signal of co-IP used to show anti-HA-HRP image.
B) Fig 8E - merged image with molecular weight markers.
C) Fig 8F - chemiluminescent signal of co-IP used to show anti-HA-HRP image.
D) Fig 8F - merged image with molecular weight markers.
E) Fig 8G - chemiluminescent signal of co-IP used to show anti-HA-HRP image.
F) Fig 8G - merged image with molecular weight markers.

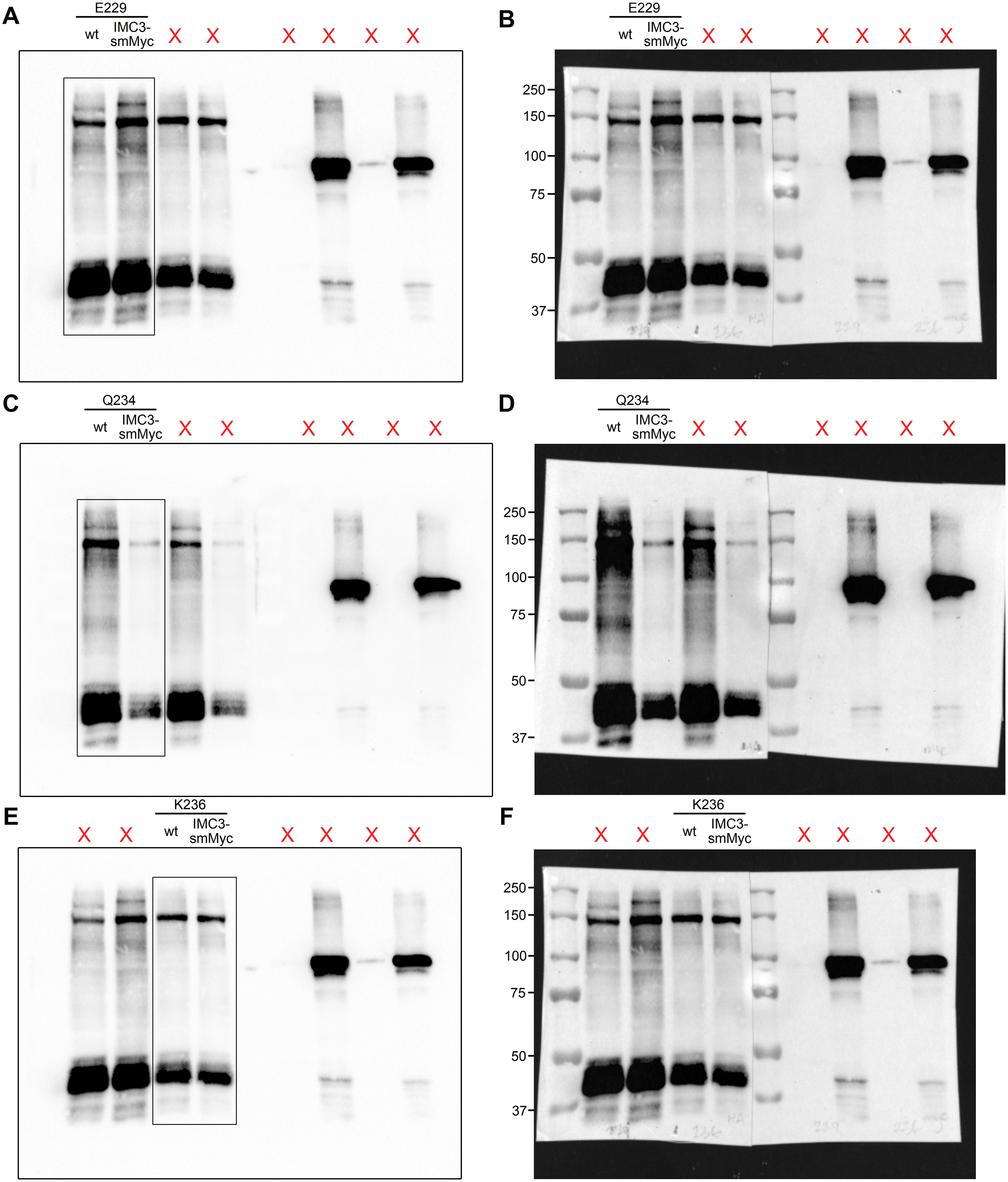

Fig 8. Western blots of IMC3-tagged and untagged amber mutants after crosslinking.
Box denotes regions used in figure. X denotes lanes unrelated to this figure.
A) Fig 8H - chemiluminescent signal of co-IP used to show anti-HA-HRP image.
B) Fig 8H - merged image with molecular weight markers.
C) Fig 8I - chemiluminescent signal of co-IP used to show anti-HA-HRP image.
D) Fig 8I - merged image with molecular weight markers.
E) Fig 8J - chemiluminescent signal of co-IP used to show anti-HA-HRP image.
F) Fig 8J - merged image with molecular weight markers.

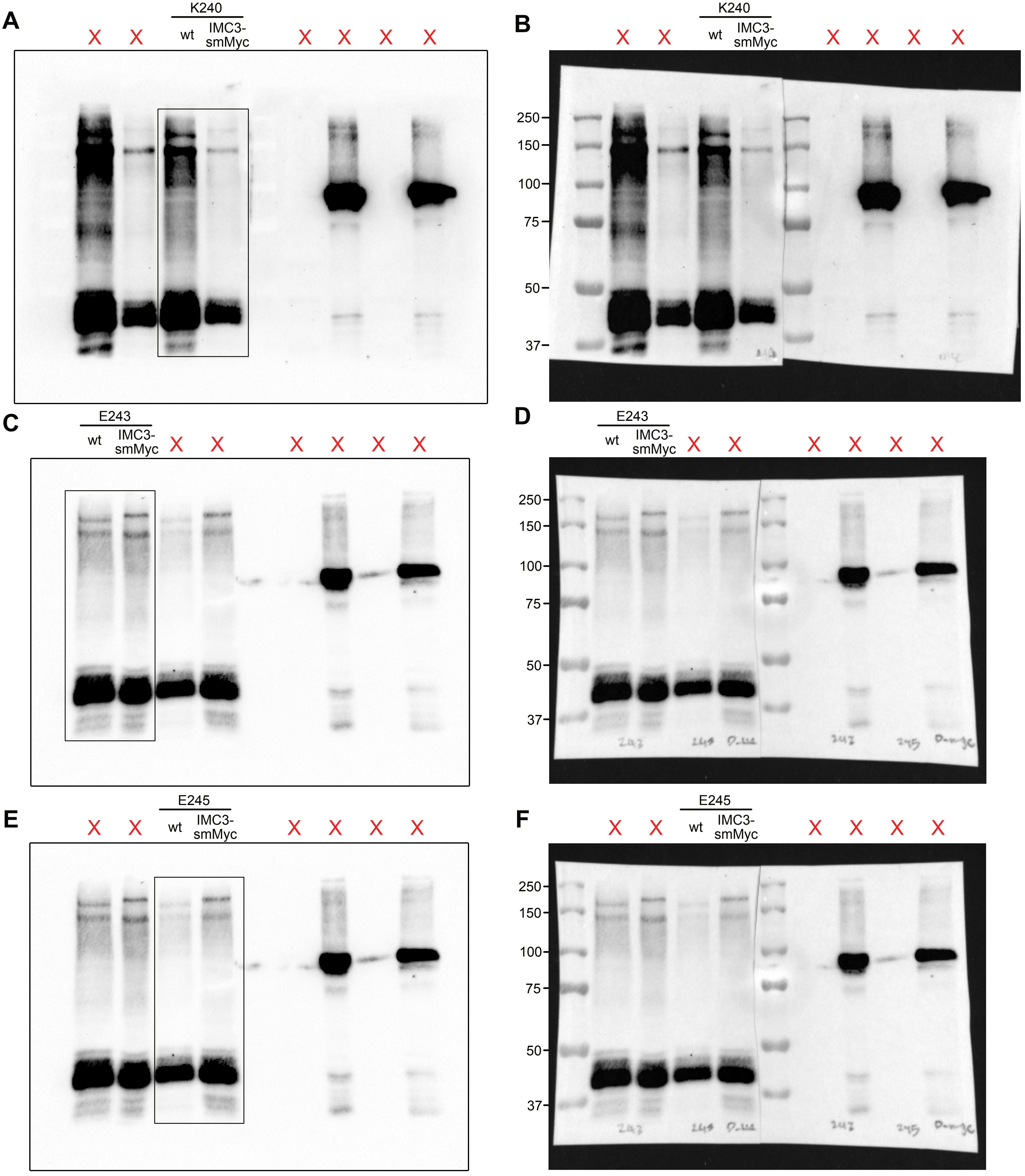

Fig 8. Western blots of IMC3-tagged and untagged amber mutants after crosslinking.
Box denotes regions used in figure. X denotes lanes unrelated to this figure.
A) Fig 8K - chemiluminescent signal of co-IP used to show anti-HA-HRP image.
B) Fig 8K - merged image with molecular weight markers.
C) Fig 8L - chemiluminescent signal of co-IP used to show anti-HA-HRP image.
D) Fig 8L - merged image with molecular weight markers.

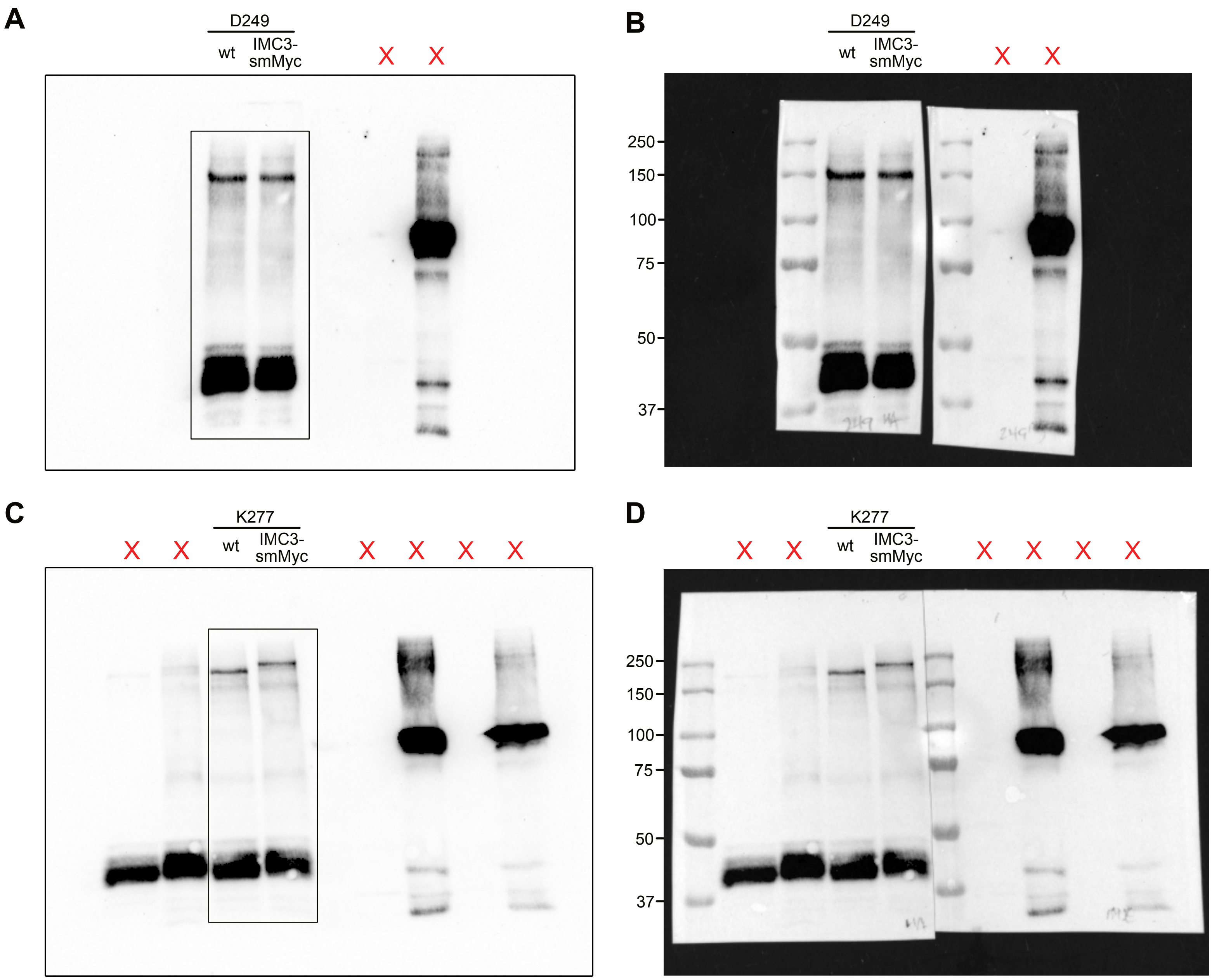

Fig 9. Western blots of ILP1-tagged and untagged amber mutants after crosslinking.
Box denotes regions used in figure. X denotes lanes unrelated to this figure.
A) Fig 9A - chemiluminescent signal of co-IP used to show anti-HA-HRP image.
B) Fig 9A - merged image with molecular weight markers.
C) Fig 9B - chemiluminescent signal of co-IP used to show anti-HA-HRP image.
D) Fig 9B - merged image with molecular weight markers.
E) Fig 9C - chemiluminescent signal of co-IP used to show anti-HA-HRP image.
F) Fig 9C - merged image with molecular weight markers.

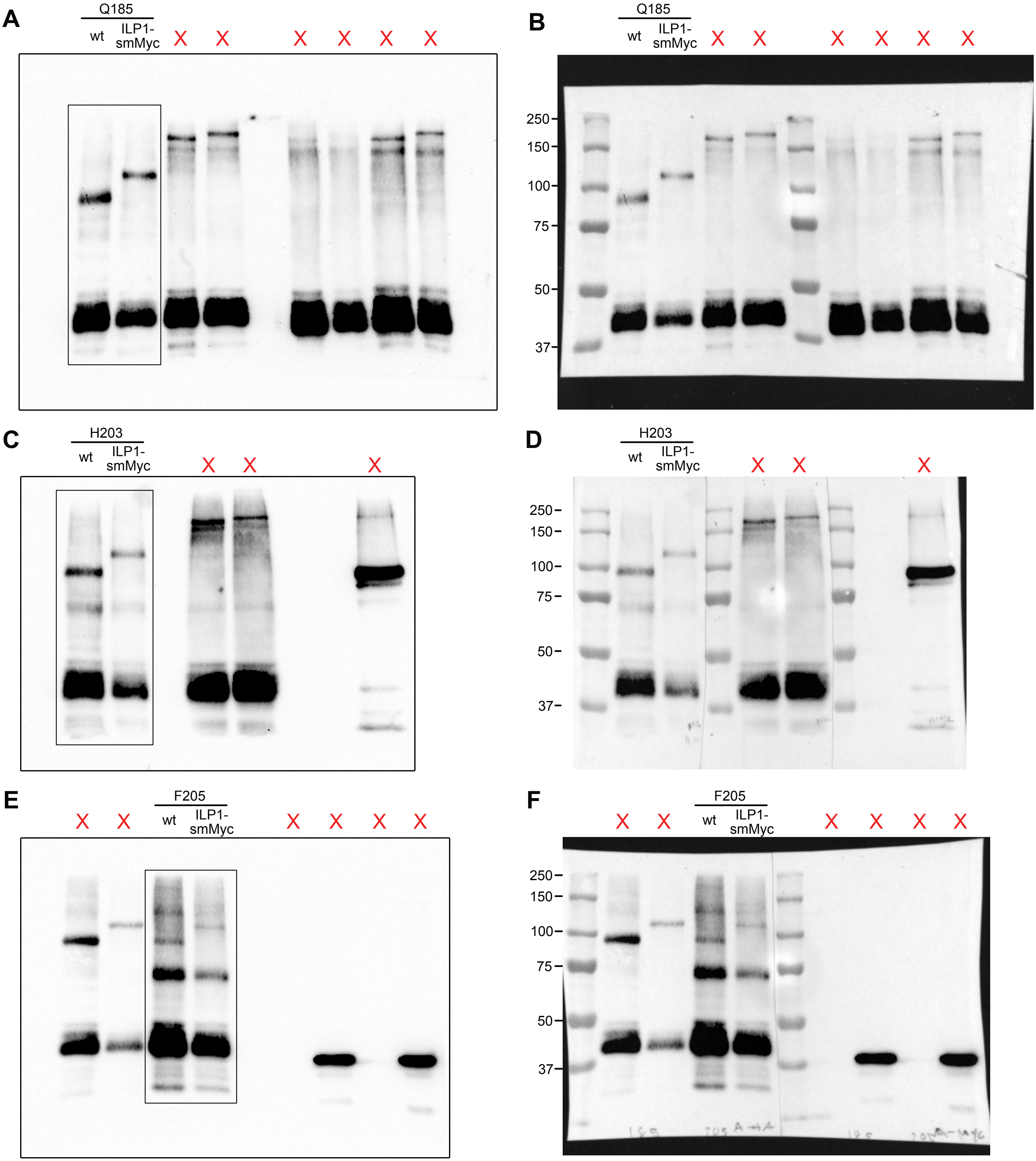

Fig S3A. Western blot of microneme secretion assay.
Box denotes regions used in figure.

A) Chemiluminescent signal used for anti-MIC2-HRP.
B) Chemiluminescent signal used for anti-GRA39-HRP.
C) Merged with colorimetric image showing molecular weight markers in kDa.

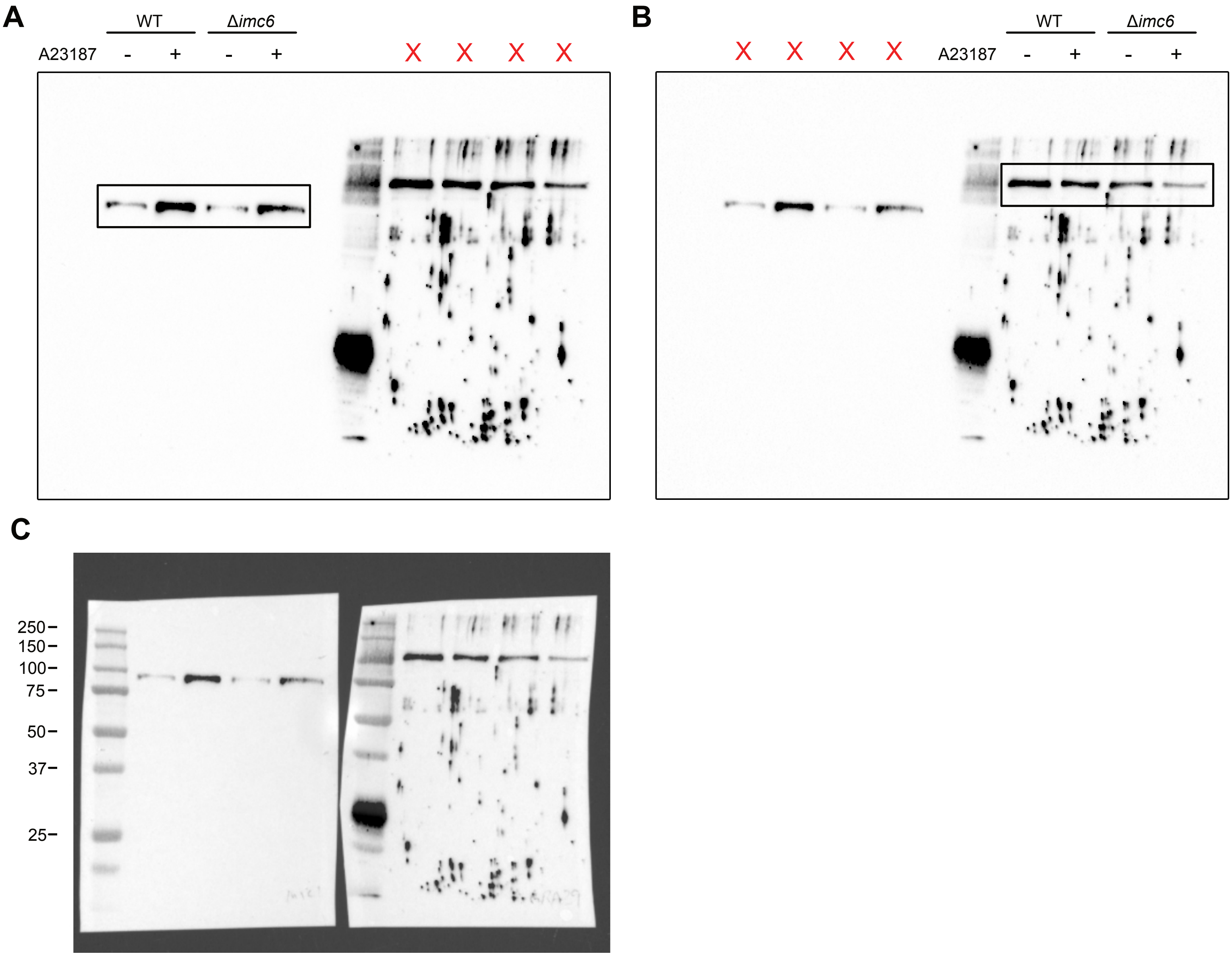

Supplement: S1 Raw Images — (PDF) [file pbio.3002809.s018.pdf]
